# Wu-Tou Decoction in Rheumatoid Arthritis: Integrating Network Pharmacology and *In Vivo* Pharmacological Evaluation

**DOI:** 10.3389/fphar.2017.00230

**Published:** 2017-05-03

**Authors:** Qingqing Guo, Kang Zheng, Danping Fan, Yukun Zhao, Li Li, Yanqin Bian, Xuemei Qiu, Xue Liu, Ge Zhang, Chaoying Ma, Xiaojuan He, Aiping Lu

**Affiliations:** ^1^Institute of Basic Research in Clinical Medicine, China Academy of Chinese Medical SciencesBeijing, China; ^2^Institute for Advancing Translational Medicine in Bone and Joint Diseases, School of Chinese Medicine, Hong Kong Baptist UniversityKowloon Tong, Hong Kong; ^3^School of Basic Medical Sciences, Shanghai University of Traditional Chinese MedicineShanghai, China; ^4^School of Life Science and Engineering, Southwest Jiaotong UniversityChengdu, China

**Keywords:** Wu-Tou decoction, rheumatoid arthritis, mechanism of action, network pharmacology, CCR5 signaling pathway in macrophages

## Abstract

**Purpose:** This study aimed to explore underlying action mechanism of Wu-Tou decoction (WTD) in rheumatoid arthritis (RA) through network pharmacology prediction and experimental verification.

**Methods:** Chemical compounds and human target proteins of WTD as well as RA-related human genes were obtained from TCM Database @ Taiwan, PubChem and GenBank, respectively. Subsequently, molecular networks and canonical pathways presumably involved in the treatment of WTD on RA were generated by ingenuity pathway analysis (IPA) software. Furthermore, experimental validation was carried out with MIP-1β-induced U937 cell model and collagen induced arthritis (CIA) rat model.

**Results:** CCR5 signaling pathway in macrophages was shown to be the top one shared signaling pathway associated with both cell immune response and cytokine signaling. In addition, protein kinase C (PKC) δ and p38 in this pathway were treated as target proteins of WTD in RA. *In vitro* experiments indicated that WTD inhibited MIP-1β-induced production of TNF-α, MIP-1α, and RANTES as well as phosphorylation of CCR5, PKC δ, and p38 in U937 cells. WTD treatment maintained the inhibitory effects on production of TNF-α and RANTES in MIP-1β-induced U937 cells after CCR5 knockdown. *In vivo* experiments demonstrated that WTD ameliorated symptoms in CIA rats, decreased the levels of IL-1β, IL-2, IL-6, TNF-α, MIP-1α, MIP-2, RANTES, and IP-10 in serum of CIA rats, as well as mRNA levels of MIP-1α, MIP-2, RANTES, and IP-10 in ankle joints of CIA rats. Furthermore, WTD also lowered the phosphorylation levels of CCR5, PKC δ and p38 in both ankle joints and macrophages in ankle joints from CIA rats.

**Conclusion:** It was demonstrated in this research that WTD played a role in inhibiting inflammatory response in RA which was closely connected with the modulation effect of WTD on CCR5 signaling pathway in macrophages.

## Introduction

Rheumatoid arthritis (RA) is a chronic and systemic autoimmune inflammatory disorder affecting multiple tissues and organs, especially for flexible joints (Chen et al., 2012). Synovial hyperplasia, inflammatory cell (including activated T and B lymphocytes, macrophages, plasma cells, mast cells, and so on) infiltration, angiogenesis, bone and cartilage erosion have been identified as the primary pathological characteristics of RA (Brzustewicz and Bryl, 2015). No effective treatment for RA is available at present. Commonly used drugs, such as non-steroidal anti-inflammatory drugs (NSAIDs), disease-modifying antirheumatic drugs (DMARDs) and glucocorticoids, have been proved to represent either limited efficacy or frequent side effects. Traditional Chinese Medicine (TCM) has been applied in treating RA for a long time in Asia, which has been gradually accepted in the world thanks to its excellent efficacy and less adverse reactions. However, the underlying mechanism by which TCM help to alleviate RA remains unknown, which hinders its further clinical application.

Wu-Tou decoction (WTD), a representative TCM formula, has been used to treat RA for over 1800 years. WTD is composed of *Aconiti Radix* (*Aconitum carmichaeli* Debx.), *Ephedrae Herba* (*Ephedra sinica* Stapf), *Astragali Radix* (*Astragalus membranaceus* Bunge var. *mongholicus* (Bunge) P.K. Hsiao), *Paeoniae Radix Alba* (*Paeonia lactiflora* Pall.) and *Glycyrrhizae Radix EtRhizoma* (*Glycyrrhiza uralensis* Fisch.). It was discovered through chemical profiling analysis that WTD mainly contained alkaloids, monoterpene glycosides, triterpene saponins, flavones and flavone glycosides (Qi et al., 2014a). As could be observed from modern pharmacological studies, WTD had significant anti-inflammatory effect. It could decrease the levels of interleukin (IL)-1β, IL-17, tumor necrosis factor (TNF)-α, vascular endothelial cell growth factor (VEGF) and prostaglandin E2 (PGE2). In addition, it could also reduce percentage of CD4^+^ cells while increase that of CD8^+^ cells in peripheral blood of rats with inflammatory arthritis (Qi et al., 2014b; Lü et al., 2015). Recently, some researchers have made great efforts to further study the underlying action mechanism of WTD taking advantage of pharmacokinetic (Zheng et al., 2012), metabolomic (Qi et al., 2014b) and systems biology methods (Zhang Y. et al., 2013). Nevertheless, comprehensive action mechanism of WTD in RA remains to be further elucidated.

Similar to other TCM formulas which are characterized by multi-component, multi-target and multi-pathway, the molecular mechanism of WTD can hardly be clarified (Corson and Crews, 2007; Zhang B. et al., 2013). TCM network pharmacology emerging recently has become a flourishing field in TCM modern studies along with the rapid progress of bioinformatics (Sharma and Sarkar, 2013; Tao et al., 2013; Liang et al., 2014). As a major tool in network pharmacology, network analysis based on widely existing databases give us access to preliminarily understand potential action mechanisms of TCM within the context of interactions at the system level. Successful attempts on TCM study have been achieved in our group by network pharmacology before this research (Liang et al., 2013; Niu et al., 2015; Zhao et al., 2015). Therefore, network pharmacology-based study was conducted in this research to explore underlying action mechanism of WTD in RA.

## Materials and Methods

The strategy of this study is available in Supplementary Figure 1.

### Network Pharmacology-Based Analysis

Firstly, chemical compounds in five herbs constituting WTD were obtained from TCM Database @ Taiwan^1^, which is the biggest TCM database around the world so far (Chen, 2011). Human protein targets of the above chemical compounds were retrieved in PubChem^2^. Besides, RA-related human genes were searched in GenBank databases^3^.

Human protein targets of WTD and RA-related human genes obtained from the first step were uploaded to IPA software. Subsequently, a set of networks were generated by IPA based on different bio-functions, in which nodes with various shapes were represented as molecules with different functions, and biological relationship between two nodes was expressed as an edge (line). In addition, the networks were ranked by scores calculated by IPA, which thereby represented the significance of molecules to networks. Furthermore, canonical pathways were obtained by means of core analysis in IPA. The significance of molecules to canonical pathways was reflected by *P*-value calculated with Fisher’s exact test. A smaller *P*-value suggested greater significance. Finally, comparison analysis was accomplished by IPA, so as to further study the action mechanism of WTD in RA.

### WTD Preparation

Wu-Tou decoction is composed of 6 g *Aconiti Radix* (*Aconitum carmichaeli* Debx.), 9 g *Ephedrae Herba* (*Ephedra sinica* Stapf), 9 g *Astragali Radix* (*Astragalus membranaceus* Bunge var. *mongholicus* (Bunge) P.K. Hsiao), 9 g *Paeoniae Radix Alba* (*Paeonia lactiflora* Pall.) and 9 g *Glycyrrhizae Radix EtRhizoma* (*Glycyrrhiza uralensis* Fisch.). All crude drugs were purchased from Beijing Tongrentang. WTD was prepared as previously described (Zhang et al., 2015). Firstly, all crude drugs were soaked in 2 L water for 2 h, then they were decocted to boiling for 1 h. The decoction was filtered through a four-layer gauze. Later, the drugs were boiled once again for 0.5 h with 2 L water and the decoction was filtered in accordance with the above method. The filtrates were merged and concentrated to a concentration of 0.75 g crude drug/mL. Subsequently, the decoction was further filtered with a 0.22 μm pore-size filter (Millipore, Billerica, MA, USA) for tests *in vitro*, and stored at 4°C for no more than 1 week. Finally, the decoction was diluted with saline for tests *in vivo* and PBS for tests *in vitro* to certain concentrations before use.

### Cell Culture

Human leukemic U937 cells were purchased from American Type Culture Collection (Manassa, VA, USA), and cultured in RPMI 1640 medium (GIBCO, Gaithersburg, MD, USA) supplemented with 10% fetal bovine serum (GIBCO, Gaithersburg, MD, USA) and 1% penicillin-streptomycin (PS) at 37°C in a humidified atmosphere with 5% CO_2_.

### Cell Viability Assay

U937 cells were planted into a 96-well cell culture plate at a density of 1.0 × 10^4^ cells/well and incubated with 10 ng/mL phorbol 12-myristate 13-acetate (PMA) (Sigma, St. Louis, MO, USA) at 37°C for 48 h in a 5% CO_2_ incubator. Subsequently, the cells were washed with PBS twice, followed by incubation with WTD (2.5–160 μg/mL) for up to 45 h at 37°C in a 5% CO_2_ incubator. 10 μL CCK-8 reagent (Dojindo, Tokyo, Japan) was then added into each well and the cells were incubated for another 3 h. Eventually, the absorbance of each well was measured at 450 nm (test wavelength) with an enzyme-linked immunosorbent assay (ELISA) reader (Bio-Tek Instruments, Winooski, VT, USA).

### WTD Treatment of U937 Cells

U937 cells were seeded in a 6-well cell culture plate at a density of 1 × 10^6^ cells/well and incubated with PMA for 48 h at 37°C. Then cells were washed for three times and treated with or without WTD at 20 μg/mL or 40 μg/mL for 2 h, followed by stimulation with 25 nM MIP-1β (PeproTech, Rocky Hill, NJ, USA) for the duration suggested in previous studies (Tomkowicz et al., 2006; Fantuzzi et al., 2008; Cheung et al., 2009). Maraviroc (MVC) (MCE, Monmouth Junction, NJ, USA), a CCR5 inhibitor, was used as the positive control at a concentration of 1 μM (Rossi et al., 2011). Cell supernatants were collected to detect levels of TNF-α, MIP-1α, and RANTES by ELISA, and the cells were harvested to detect levels of total and phosphorylated CCR5, PKC δ and p38 with western blotting.

### SiRNA Transfection

CCR5 siRNA was obtained from Dharmacon (Lafayette, CO, USA), and control siRNA was purchased from Santa Cruz Biotechnology Inc. (Delaware, CA, USA). Transfection was performed using Lipofectamine 2000 (Invitrogen Life Technologies, Carlsbad, CA, USA) in strict accordance with instructions from manufacturers. Briefly, PMA-induced U937 cells were grown to 30–50% confluence in a 6-well plate before they were transfected with Lipofectamine 2000. SiRNA and Lipofectamine 2000 were premixed for 20 min before they were added into the cells. After 24 h of transfection, the cells were incubated with or without WTD for 2 h, followed by exposure to MIP-1β for another 24 h. After that, cell supernatants were collected to detect levels of TNF-α and RANTES by ELISA.

### Animals

Male Sprague Dawley (SD) rats aged 8–10 weeks with a mean weight of 180–200 g were purchased from Research Institute of Experimental Animals, Chinese Academy of Medical Science. The rats were fed with food and water *ad libitum*, and then were allowed to acclimatize themselves for 1 week before the initiation of experiment. The rats were housed in a temperature-, humidity-, and light-controlled environment. The light-dark cycle was 12 h:12 h with the light phase being set from 06:00 to 18:00. The rodent license of the laboratory (NO.SYXK 11-00-0039) was issued by National Science and Technology Ministry of China. This study was approved by the Research Ethics Committee of Institute of Basic Theory of Chinese Medicine, China Academy of Chinese Medical Sciences.

### Induction of Collagen Induced Arthritis (CIA)

Collagen induced arthritis (CIA) animal model was constructed as previously described (Hsu et al., 2012). Male SD rats were immunized using an emulsion consisted of equivalent parts of incomplete Freund’s adjuvant (Chondrex, Redmond, WA, USA) and bovine type II collagen (C II) (Chondrex, Redmond, WA, USA). The rats received an intradermal injection of 200 μL emulsion (200 μg of bovine CII) into the base of tail on day 0. Booster doses were administered on day 7 with 100 μL injection of same emulsion being given into the base of tail. The onset of CIA could be observed between days 11 and 13 after the first immunization. The following clinical parameters were measured, including body weight and arthritis index (AI) score. AI score for each hind ankle joint was recorded by the same observer, who was blind to the treatment received by animals. Scoring was performed with a 0–4 scale (Shahrara et al., 2003), where 0 = no swelling or erythema; 1 = slight swelling and/or erythema; 2 = low-to-moderate edema; 3 = pronounced edema with limited joint usage; and 4 = excess edema with joint rigidity. The maximum score of each rat was eight.

### WTD Treatment of Animals

Methotrexate with a purity of over 99.0% was purchased from Sigma (St. Louis, MO, USA). The rats were randomly divided into five groups after the successful induction of CIA model with eight rats in each group, which were normal group (N), model group (M), WTD high dose group (WH, 7.5 g/kg/d), WTD low dose group (WL, 3.75 g/kg/d) and methotrexate group (MTX, 1 mg/kg/w). All agents were intragastrically administered at a volume of 1 mL/100 g for 4 weeks. The dosage of WTD in WL group (3.75 g crude drug/kg/d) was nearly equal to that for RA patients (42 g crude drug/person/day). The dosage of MTX was set as previously described (Le Goff et al., 2009).

### Enzyme-Linked Immunosorbent Assay (ELISA)

Cell supernatants were collected after 24 h stimulation with MIP-1β. Levels of TNF-α, MIP-1α, and RANTES in U937 cell supernatants, as well as those of IL-1β, IL-2, IL-6, TNF-α, macrophage inflammatory protein (MIP)-1α, MIP-2, regulated upon activation normal T cell expressed and secreted (RANTES) and interferon-inducible protein (IP10) in rat serum were detected with commercially available enzyme-linked immunosorbent assay (ELISA) kits (eBioscience, San Diego, CA, USA) according to manufacturer’s instructions.

### Western Blotting

Western blotting was conducted following previous method (Shahrara et al., 2003). Briefly, U937 cells were harvested after 10 min stimulation with MIP-1β. The cells and ankle joints of rats were lysed in RIPA Lysis Buffer (Beyotime, Shanghai, China) containing PMSF (Amresco, Houston, TX, USA). Ankle joint homogenization was completed on ice with a motorized homogenizer. Homogenates were centrifuged at 13000 rpm for 20 min at 4°C, filtered through a 0.45 μm pore-size Millipore filter, and stored at -80°C until use. Protein concentration in each lysate was determined using a bicinchoninic acid assay (Pierce, Rockford, IL, USA) with bovine serum albumin (Sigma, St. Louis, MO, USA) as the standard.

Equal amount of each sample was subjected to 10% sodium dodecyl sulfate-polyacrylamide gel electrophoresis and transferred to nitrocellulose membranes utilizing a wet transblotting apparatus. Nitrocellulose membranes were blocked with 3% bovine serum albumin in Tris buffered saline-Tween 20 (TBST) buffer (20 mM Tris, 137 mM NaCl, pH 7.6, with 0.1% Tween 20) for 30 min at room temperature. Blots were incubated for 10 min at room temperature followed by overnight at 4°C and 30 min at room temperature on the 2nd day with anti-p-CCR5, anti-p-PKC δ, anti-p-p38 antibody at 1:1000 and anti-CCR5, anti-PKC δ at 1:5000, anti-p38 (Abcam, Cambridge, UK) at 1:4,000 in TBST containing 3% bovine sera albumin. After that, blots were washed five times and then incubated in horseradish peroxidase–conjugated antibody (1:10,000 dilution in TBST containing 5% non-fat milk) for 40 min at room temperature. All blots were developed with enhanced chemiluminescence reagents based on instructions from manufacturers. Blots were scanned and analyzed for measurement of the band intensities with UN-SCAN-IT version 5.1 software. Band intensity was calculated as follows: band intensity = sum of all pixel values in the segment selected - background pixel value in that segment.

### Real-Time PCR

Ankle joints were dissected from rats, snap-frozen in liquid nitrogen, grounded into powder, and homogenized under RNase-free conditions. RNA isolation and real-time PCR assay were carried out following the protocol in previous study (Liu et al., 2013). Briefly, total RNA was extracted from the tissue homogenate with TRIzol reagent (Invitrogen, Carlsbad, CA, USA) according to manufacturer’s instructions. The total RNA (1 μg) was reverse transcribed to cDNA using PrimeScript^TM^ RT reagent Kit with gDNA Eraser (TaKaRa, Kusatsu, Japan) in accordance with instruction manual. The specific transcripts were quantified through the method of quantitative real-time PCR using SYBR^®^Premix Ex Taq^TM^ II (Tli RNaseH Plus) with ROX plus (TaKaRa, Kusatsu, Japan). And they were analyzed by ABI 7500 real-time PCR system (AppliedBiosystems, Foster, CA, USA). Gene-specific primers were synthesized by Invitrogen and the following primer sequences were used: TGCTGCTTCTCCTATGGACG (forward) and GCTCAGTGATGTATTCTTGGACC (reverse) for MIP-1α, CAGAGCTTGAGTGTGACG (forward) and TCGTACCTGATGTGCCTC (reverse) for MIP-2, CCCTGCTGCTTTGCCTACCT (forward) and ACACTTGGCGGTTCCTTCG (reverse) for RANTES, GCACCTGCATCGACTTCCAT (forward) and TTCTTTGGCTCACCGCTTTC (reverse) for IP-10, and TGGAGTCTACTGGCGTCTT (forward) and TGTCATATTTCTCGTGGTTCA (reverse) for GAPDH. The mRNA levels were normalized to GAPDH mRNA level. PCR conditions were shown as below: at 95°C for 30 s, 45 cycles at 95°C for 5 s and at 60°C for 40 s. Relative mRNA expression was calculated by comparative CT method.

### Histological Examination

Ankle joints of rats were fixed in 4% paraformaldehyde, decalcified in 10% EDTA bone decalcifier, and embedded in paraffin. Four-micrometer sections were prepared and stained with H&E for histological examination. Histopathological characteristics were evaluated blindly as described (Kok et al., 2011), where grade 1 = hyperplasia of the synovial membrane and presence of polymorphonuclear infiltrates; grade 2 = pannus and fibrous tissue formation as well as focal subchondral bone erosion; grade 3 = articular cartilage destruction and bone erosion; and grade 4 = extensive articular cartilage destruction and bone erosion.

### Immunohistochemistry

The sections were dewaxed and hydrated using xylene and a graded series of alcohols. Activity of endogenous peroxidase was quenched with 3% H_2_O_2_. Then the tissues were incubated with anti-p-CCR5, anti-p-PKC δ, and anti-p-p38 (Abcam, Cambridge, UK) overnight at 4°C, followed by incubation with Polink-2 plus^®^ Polymer HRP Detection System (ZSGB-BIO, Beijing, China) according to instructions from manufacturers. Final color product was developed with DAB Kit (ZSGB-BIO, Beijing, China). Later, sections were counterstained with hematoxylin (Leagene, Beijing, China). Meanwhile, PBS was used for control staining instead of primary antibodies. Images were captured using a LEICA DFC300 FX (Leica microsystems Ltd) attached to LEICA DM6000B at a magnification of 200×.

Immunohistochemical semi-quantitative analysis was performed as previously described (Zhang et al., 2015). Uncounterstained specimens were examined utilizing a Leica image analyzer and analyzed by computer image analysis (Leica QWin V3) in a blind manner. Ten digital images were recorded for each specimen from ankle joints so as to localize and identify areas with positively stained cells, and quantitative analysis was performed according to the color cell separation. The results were expressed as the mean region of interest, which represented the percentage of area covered with positively stained cells per image at a magnification of 200×.

### Immunofluorescent Staining

To further detect phosphorylation levels of CCR5, PKC δ, and p38 in macrophages in ankle joints, immunofluorescent staining was performed according to previous study (Ma et al., 2011). Sections were treated with 10% bovine serum albumin for 1 h at 24°C, and then incubated with each primary antibody for 18 h at 4°C. The primary antibodies used were rabbit polyclonal anti-p-CCR5, anti-p-PKCδ and anti-p-p38 mixed with mouse monoclonal anti-CD68. After being washed with PBS, the sections were incubated with a mixture of FITC-conjugated and rhodamine-conjugated secondary antibodies for 1 h at 24°C, followed by incubation with DAPI for 10 min at 24°C. Sections were mounted in the anti-fading medium Vectashield after being washed. Samples were examined using a confocal laser scanning microscope, LSM510MET (Carl Zeiss, Jena, Germany).

### Statistical Analysis

Statistical analysis was performed with SPSS 18.0 software. All data were expressed as mean ± SD. Comparisons of numerical data between two groups were calculated by Student *t*-tests. Differences in mean values of various groups were analyzed by ANOVA. Difference with *P*-value < 0.05 was considered as statistically significant.

## Results

### Results of Network Pharmacology-Based Analysis

**FIGURE 1:**
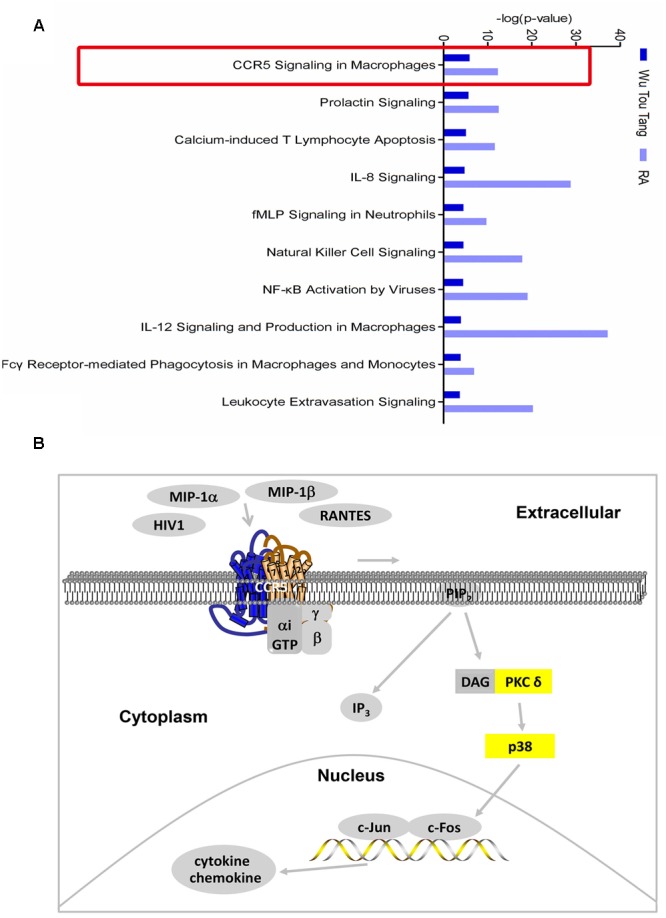
**Shared signaling pathways of WTD and RA. (A)** Top 10 shared signaling pathways of Wu-Tou decoction (WTD) and RA associated with cell immune response and cytokine signaling. Top 1 signaling pathway (red rectangle marked) was the one we focused on. **(B)** CCR5 signaling pathway in macrophages. Molecules in yellow were targets of WTD in RA.

One hundred and seventy-four chemical compounds (Supplementary Table 1) and 186 human target proteins (Supplementary Table 2) of WTD were obtained in this study. Molecular networks constructed by RA-related genes or targets of WTD could be seen in Supplementary Tables 3, 4. The top 3 of them were shown in Supplementary Figures 2, 3, respectively. The top 12 bio-functions regulated by both targets of WTD and RA-related genes were shown in Supplementary Figure 4. The results indicated that the action mechanism of WTD in RA might be related to its regulation effect on inflammatory response, hematological system development and function, cell-to-cell signaling and interaction, and so on. Furthermore, 346 and 394 signaling pathways of WTD and RA were obtained, respectively. Among which, 314 signaling pathways were shared by WTD and RA. The top 10 shared signaling pathways associated with cell immune response and cytokine signaling were shown in **Figure 1A**. The top 1 shared signaling pathway, CCR5 signaling pathway in macrophages, was focused for further study. It could be seen from **Figure 1B** that ligands of CCR5, such as HIV1, MIP-1α, MIP-1β, and RANTES, could interact with CCR5 and activate CCR5 signaling pathway. PKC δ and p38 (yellow molecules) in this signaling pathway were shown as targets of WTD in RA. Besides, the signaling pathway could ultimately regulate the expression of chemoattractants and proinflammatory mediators.

### Inhibiting the Production of Cytokines and Chemokines in MIP-1β-induced U937 Cells by WTD

**FIGURE 2:**
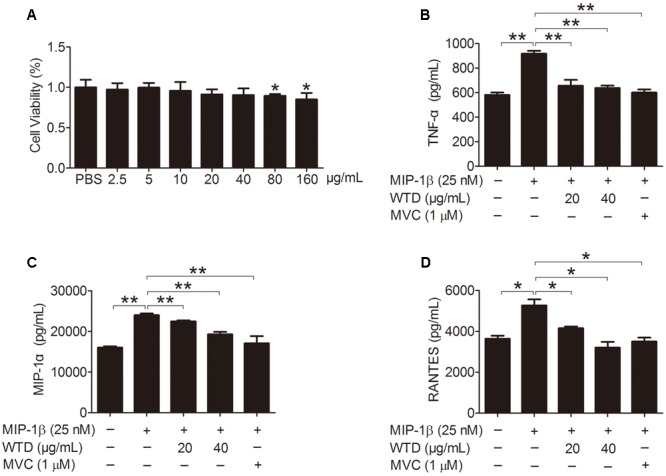
**Inhibition of MIP-1β-induced production of TNF-α, MIP-1α, and RANTES by WTD in U937 cells. (A)** Effect of WTD on cell viability of U937. U937 cells (1 × 10^4^ cells/well in a 96-well plate) were pretreated with different concentrations of WTD for 45 h, followed by incubation with 10 μL CCK8 for 3 h. And then OD values were measured at 450nm. ^∗^*P* < 0.05 vs. PBS group. **(B–D)** Inhibition the production of TNF-α, MIP-1α, and RANTES in MIP-1β-induced U937 cells by WTD. PMA-induced U937 cells were pretreated with or without WTD, followed by exposure to MIP-1β (25 nM) for 24 h. The cell supernatants were collected to detect the levels of TNF-α, MIP-1α, and RANTES by ELISA. Data were expressed as mean ± SD. ^∗^*P* < 0.05, ^∗∗^*P* < 0.01.

Firstly, the effect of WTD on the viability of U937 cells was evaluated through CCK8 assay, which demonstrated that WTD (2.5–40 μg/mL) had no effect on the viability of U937 cells at 48 h (**Figure 2A**). Stimulation of U937 with MIP-1β (25 nM) for 24 h resulted in significant increase of TNF-α, MIP-1α, and RANTES, as compared with untreated control. Pretreatment with WTD (20 and 40 μg/mL) led to significant inhibition of MIP-1β-induced TNF-α, MIP-1α and RANTES production (**Figures 2B–D**).

### Effects of WTD on Levels of CCR5, PKC δ, and P38 in MIP-1β-induced U937 Cells

**FIGURE 3:**
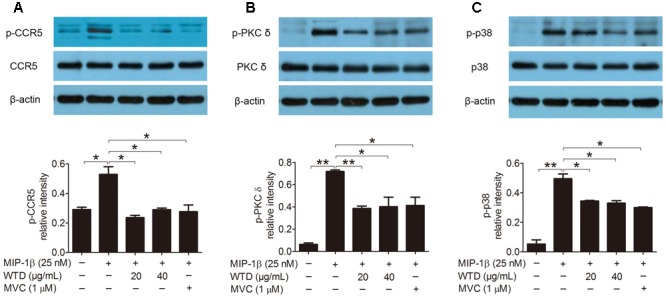
**Inhibition of total and phosphorylation levels of CCR5, PKC δ, and p38 in MIP-1β-induced U937 cells by WTD.** Cells were pre-treated with PMA for 48 h and then incubated with or without WTD or MVC for 2 h. Subsequently, cells were stimulated with MIP-1β (25 nM). After 10 min of stimulation, cells were collected to detect total and phosphorylated (p) CCR5 **(A)**, PKC δ **(B)**, and p38 **(C)** by western blotting. ^∗∗^*P* < 0.01, ^∗^*P* < 0.05.

Then the effects of WTD on the expression of CCR5, PKC δ and p38 as well as their phosphorylation levels in MIP-1β-induced U937 cells were investigated. As could be seen from **Figures 3A–C**, MIP-1β stimulation significantly increased the phosphorylation levels of CCR5, PKC δ, and p38 (*P* < 0.05 or *P* < 0.01), with no effect on their total level. Both WTD and MVC could significantly reduce the phosphorylation levels of CCR5, PKC δ, and p38 in MIP-1β-induced U937 cells (*P* < 0.05 or *P* < 0.01). These findings indicated that WTD could modulate CCR5 signaling pathway in MIP-1β-induced U937 cells.

### CCR5 Signaling Pathway Might Be Involved in the Regulation Effects of WTD on MIP-1β-Induced Production of Cytokines and Chemokines

**FIGURE 4:**
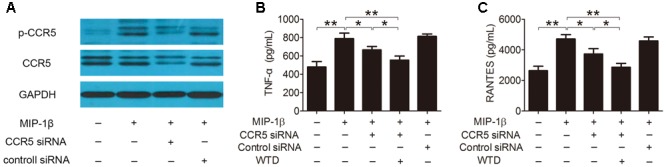
**Effects of WTD on production of TNF-α and RANTES in MIP-1β-induced U937 cells after CCR5 knockdown.** U937 cells were transfected with or without CCR5 siRNA, then treated with or without WTD for 2 h, and then exposed to MIP-1β (25 nM) for 24 h. Total CCR5 and its phosphorylation form **(A)** were detected by western blotting. The levels of TNF-α **(B)** and RANTES **(C)** in cell supernatants were detected by ELISA. ^∗∗^*P* < 0.01, ^∗^*P* < 0.05.

Furthermore, the role of CCR5 signaling pathway in the modulation effects of WTD on MIP-1β-induced production of cytokines and chemokines was further studied. As was shown in **Figure 4A**, the expression of total and phosphorylated CCR5 was significantly lowered in MIP-1β-induced U937 cells transfected with CCR5 siRNA compared to untransfected cells. CCR5 knockdown inhibited MIP-1β-induced production of TNF-α and RANTES. Much stronger inhibitory effects on the production of TNF-α and RANTES could be seen in MIP-1β-induced U937 cells after combined treatment with CCR5 siRNA and WTD in comparison with treatment with CCR5 siRNA alone (*P* < 0.05) (**Figures 4B,C**).

### WTD Ameliorated Symptoms of CIA Rats

**FIGURE 5:**
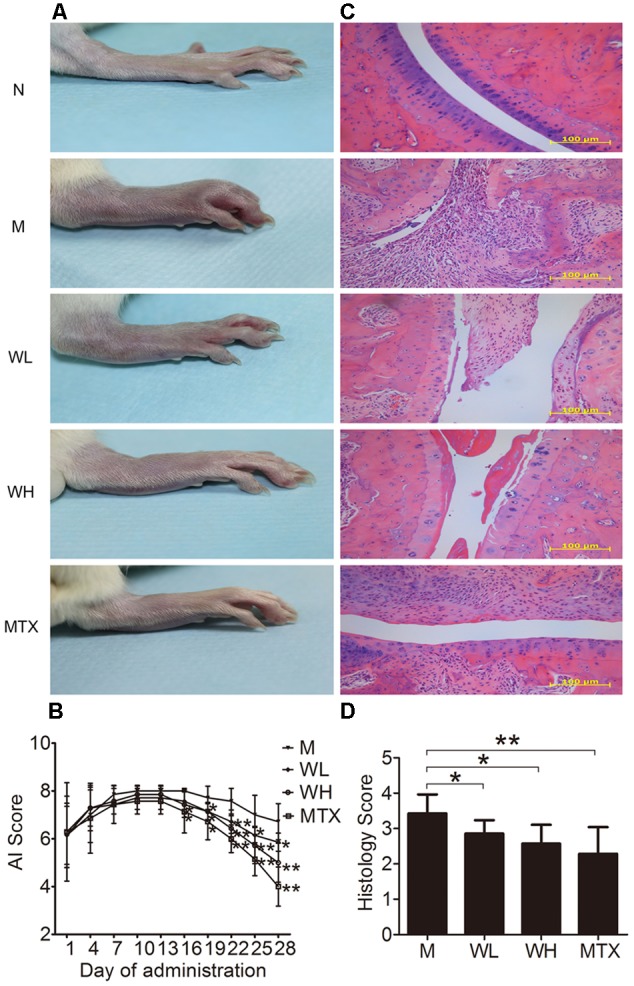
**Effects of WTD on arthritis severity of CIA rats. (A)** Representative photograph of ankle joint from each group. These photos were taken on the last day of treatment. **(B)** AI score of each group. ^∗^*P* < 0.05, ^∗∗^*P* < 0.01 vs. model group. **(C)** Representative histological finding of ankle joint from each group. Tissue sections from ankle joints were stained with H&E. Original magnification 200×. **(D)** Histological score in each group. ^∗^*P* < 0.05, ^∗∗^*P* < 0.01 vs. model group. Abbreviations: N, normal group; M, model group; WL, Wu-Tou decoction low dose group (3.75 g/kg/d); WH, Wu-Tou decoction high dose group (7.5 g/kg/d); MTX, methotrexate group (1 mg/kg/w).

Collagen induced arthritis rats were used in the study to verify the therapeutic effect of WTD on RA. Morphological features of arthritis, including erythema and swelling, could be markedly observed in model group, while WTD treatment relieved arthritis severity in CIA rats (**Figure 5A**). Consistently, AI scores in WTD-treated CIA rats were significant lower than those in vehicle-treated CIA rats (**Figure 5B**). Histopathological results (**Figure 5C**) indicated the presence of massive influx of inflammatory cells, synovial hyperplasia, and severe erosion of cartilage and bone in ankle joints of CIA rats. Whereas, symptoms of inflammation, synovial hyperplasia and joint destruction in rats treated with WTD were remarkably alleviated. Histological score analysis also manifested that WTD could prominently inhibit the histopathological changes in ankle joints of CIA rats (**Figure 5D**). All these results proved the therapeutic effect of WTD on RA.

### Effects of WTD on Production of Cytokines and Chemokines in CIA Rats

**FIGURE 6:**
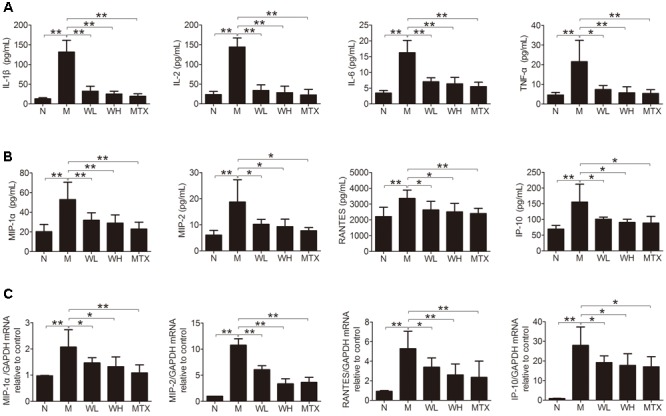
**Effects of WTD on production of cytokines and chemokines in CIA rats. (A)** Effect of WTD on production of cytokines. The levels of IL-1β, IL-2, IL-6, and TNF-α in serum of rats were determined by ELISA. **(B)** Effect of WTD on production of chemokines. Levels of MIP-1α, MIP-2, RANTES, and IP-10 in serum of rats were determined by ELISA. **(C)** Effect of WTD on mRNA levels of chemokines. The mRNA levels of MIP-1α, MIP-2, RANTES, and IP-10 in rats’ ankle joints were detected by real-time PCR. ^∗^*P* < 0.05, ^∗∗^*P* < 0.01.

Several cytokines and chemokines were detected by ELISA and Real-time PCR, so as to observe the effects of WTD on production of proinflammatory cytokines and chemokines in CIA rats. The results showed that WTD treatment dramatically decreased the serum levels of IL-1β, IL-2, IL-6, TNF-α, MIP-1α, MIP-2, RANTES, and IP-10 in CIA rats (**Figures 6A,B**). Meanwhile, the mRNA levels of MIP-1α, MIP-2, RANTES, and IP-10 in ankle joints of WTD-treated CIA rats also decreased significantly in comparison with those in vehicle-treated CIA rats (**Figure 6C**).

### Effects of WTD on Levels of CCR5, PKC δ and P38 in Ankle Joints of CIA Rats

**FIGURE 7:**
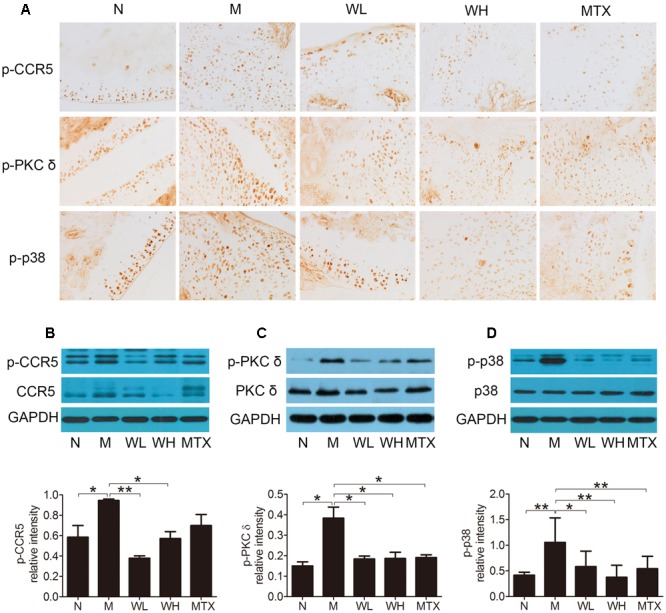
**Effects of WTD on level of total and phosphorylated CCR5, PKC δ, and p38 in ankle joints of CIA rats. (A)** Immunohistochemistry findings of p-CCR5, p-PKC δ, and p-p38 in each group. Tissue sections from ankle joints in each group were stained with anti-p-CCR5, anti-p-PKC δ, and anti-p-p38 or isotype control Abs. The cells stained with each Ab were shown in brown. Original magnification 200×. **(B–D)** Western blotting results of total and phosphorylated CCR5, PKC δ, and p38. *^∗^P* < 0.05, *^∗∗^P* < 0.01.

The expression of CCR5, PKC δ, and p38, as well as their phosphorylation levels in ankle joints was detected by immunohistochemistry and western blotting, so that the action mechanism of WTD in CIA rats could be investigated. The results could be seen in **Figures 7A–D**, which showed that WTD significantly decreased the phosphorylation levels of CCR5, PKC δ and p38 in ankle joints of CIA rats (*P* < 0.05 or *P* < 0.01). In addition, WTD also decreased total level of CCR5 in CIA rat’s ankle joint, but had no effect on total PKC δ and p38.

### Effects of WTD on Phosphorylation Levels of CCR5, PKC δ, and P38 in Macrophages in Ankle Joints of CIA Rats

**FIGURE 8:**
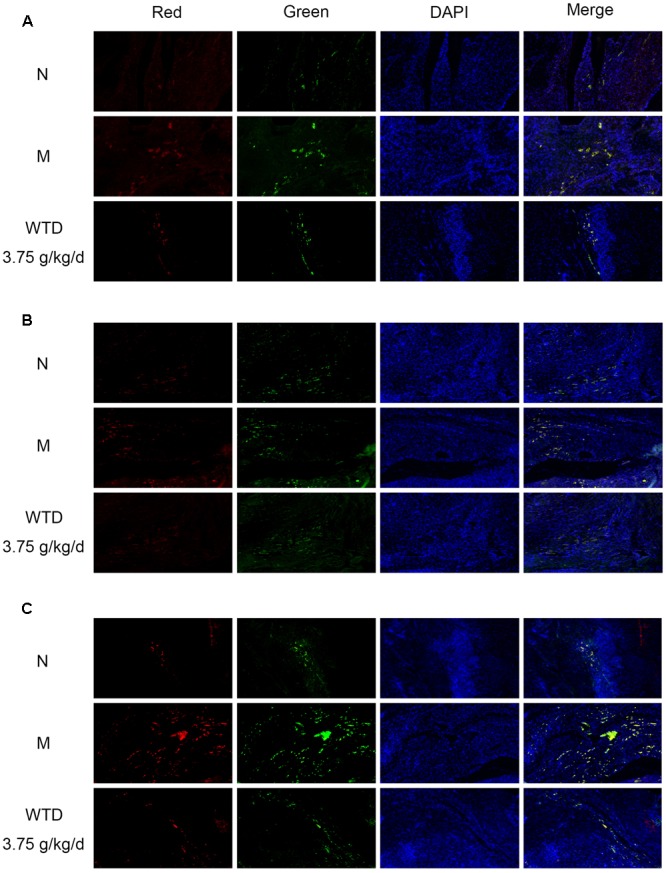
**Effects of WTD on phosphorylation levels of CCR5, PKC δ, and p38 in macrophages in ankle joints of CIA rats.** Representative immunofluorescence images of p-CCR5 **(A)**, p-PKC δ **(B)**, p-p38 **(C)** (red) and CD68 (green). Original magnification 100×.

Phosphorylation levels of CCR5, PKC δ, and p38 in macrophages in ankle joints were detected by immunofluorescence, so as to further study the effect of WTD on CCR5 signaling pathway in macrophages in CIA rats. It could be observed in **Figures 8A–C** that the levels of p-CCR5, p-PKC δ, and p-p38 in CD68^+^ cells infiltrated in the ankle joints of vehicle-treated CIA rats were significantly higher than those in normal rats. And higher levels of p-CCR5, p-PKC δ, and p-p38 were inhibited significantly by WTD.

## Discussion

Network pharmacology is regarded as a promising method for complicated mechanism study of TCM and new drug discovery (Hopkins, 2008; Wei et al., 2016). Multiple canonical pathways closely associated with RA, such as G-protein coupled receptor signaling pathway, IL-8 signaling pathway, as well as IL-12 signaling and production pathway in macrophages, were discovered by means of network pharmacology in the study. These findings were consistent with those in previous studies (Kang et al., 2000; Neumann et al., 2014; Valcamonica et al., 2014). Meanwhile, it was discovered that CCR5 signaling pathway in macrophages was the top 1 shared signaling pathway of WTD and RA. However, there is no study regarding the effects of WTD on CCR5 signaling pathway in macrophages so far.

As we all know, massive inflammatory infiltration is the most notable characteristics in RA. (Koch et al., 1994; Shadidi, 2004; Stanczyk et al., 2005; Pavkova Goldbergova et al., 2012; Tanaka et al., 2013; Antonelli et al., 2014). It is reported that 40% or more of the inflammatory infiltration can be attributed to macrophages which are heavily recruited to the inflammatory sites as the progress of RA (Asquith et al., 2015). C-C chemokine receptor type 5 (CCR5), one of the G protein-coupled receptors, is highly expressed in macrophages. The connection of CCR5 with its ligands, such as MIP-1α, MIP-1β, and RANTES, plays a vital role in chemotaxis of macrophages (Tomkowicz et al., 2006). As was demonstrated in this study, total level of CCR5 in ankle joint of CIA rat was significantly higher than that in normal rat, which was consistent with previous studies (Haringman et al., 2006; Duan et al., 2014). Higher CCR5 level in morbid joint than normal joint can be attributed to increased expression of CCR5 in CCR5^+^ cells or/and increased number of CCR5^+^ cells in morbid joint. Furthermore, WTD reduced the total level of CCR5 in ankle joint of CIA rat, but had no effect on total level of CCR5 in MIP-1β-induced U937 cells. It was speculated that WTD might inhibit the accumulation of CCR5^+^ cells in ankle joint of CIA rat. In addition, some chemical compounds in WTD could be catabolized by human body, and these metabolites might also exert pharmacological action. This might be one of the reasons for which WTD showed different effect on total level of CCR5 in *in vivo* and *in vitro* tests. Certainly, further studies are needed to confirm these speculations.

The connection of CCR5 with its ligands not only leads to chemotaxis of macrophages, but also induces the production of inflammatory mediators in macrophages (Choe et al., 2001; Fantuzzi et al., 2008). It has been proved that activated macrophages can release various cytokines (such as TNF-α, IL-6) and chemokines (such as MCP-1, MIP-1α, MIP-1β, and RANTES) during the initiation of inflammatory process (Fantuzzi et al., 2008; Valledor et al., 2010). Consistently, increased production of some cytokines and chemokines in MIP-1β-induced U937 cells was also observed in our *in vivo* tests, which was inhibited by WTD treatment. Moreover, our *in vivo* tests suggested that increased levels of IL-1β, IL-2, IL-6, TNF-α, MIP-1α, MIP-2, RANTES, and IP-10 in serum of CIA rat, as well as increased mRNA levels of MIP-1α, MIP-2, RANTES, and IP-10 in ankle joint of CIA rat was also reduced by WTD.

The underlying mechanism by which WTD inhibit inflammatory response was further studied. As shown in **Figure 1B**, PKC δ and p38 that involved in CCR5 signaling pathway in macrophages were predicted as targets of WTD. As reported in previous literature, PKC δ and p38 could promote the expression of cytokines and chemokines in macrophages (Higai et al., 2008; Tang et al., 2010; Si et al., 2012; Ren et al., 2014; Park et al., 2015). Then, *in vitro* and *in vivo* tests were designed to validate the effect of WTD on CCR5 signaling pathway in macrophages. Results of *in vitro* experiments indicated that WTD could suppress MIP-1β-induced phosphorylation levels of CCR5, PKC δ, and p38 in U937 cells with no effect on total levels of CCR5, PKC δ, and p38. In addition, WTD could lower phosphorylation levels of CCR5, PKC δ, and p38 in ankle joints of CIA rats. Furthermore, the immunofluorescence result suggested that WTD could decrease phosphorylation levels of CCR5, PKC δ, and p38 in macrophages in ankle joints of CIA rats.

Furthermore, as we listed in Supplementary Table 2, the chemical compounds in WTD targeting PKC δ and p38 were glycyrrhetinic acid and phenethylcaffeate, respectively. Both of them are small molecule compounds. Glycyrrhetinic acid, one of the active components of *Glycyrrhizae Radix Et Rhizoma* (*Glycyrrhiza uralensis* Fisch.), have excellent anti-inflammatory effect (Wang et al., 2011). Moreover, it has been reported that glycyrrhetinic acid could not only potentiate the therapeutic effects but could also decrease the adverse effects of NSAIDs or DMARDs during treatment course of RA (Huang et al., 2016). Phenethylcaffeate, one of the active components of *Ephedrae Herba* (*Ephedra sinica* Stapf), exert multiple pharmacological effects, including anti-inflammation, immunomodulation (Armutcu et al., 2015; Li et al., 2017), regulation of VEGF-induced angiogenesis (Chung et al., 2013), inhibition of leukotrienes biosynthesis (Boudreau et al., 2012), and so on. All of these studies indicated that glycyrrhetinic acid and phenethylcaffeate might be served as additives to RA treatment.

By way of conclusion, the underlying action mechanism of WTD in RA was studied in this research by combining network pharmacology prediction and experimental validation. This study provided a practicable and effective strategy for complicated mechanism study of TCM, and might be conducive to new drug discovery.

## Conclusion

This study demonstrated that WTD could inhibit the inflammatory response in RA which was closely connected with the modulation effect of WTD on CCR5 signaling pathway in macrophages.

## Author Contributions

QG contributed to the conception and design of the study, the acquisition of data, the analysis and interpretation of the data and drafting of the work. KZ, DF, YZ, LL, YB, XL, XQ, and GZ contributed to the acquisition of data and the analysis and interpretation of the data. XH contributed to the conception and design of the study and revising the work. CM and AL contributed to final approval of the version to be published.

## Conflict of Interest Statement

The authors declare that the research was conducted in the absence of any commercial or financial relationships that could be construed as a potential conflict of interest.

## Footnotes

1 http://tcm.cmu.edu.tw/

2 https://www.ncbi.nlm.nih.gov/pccompound/

3 http://www.ncbi.nlm.nih.gov/genbank/
