# Supplementary material for: Wu-Tou Decoction in Rheumatoid Arthritis: Integrating Network Pharmacology and In Vivo Pharmacological Evaluation
Source: Front Pharmacol. 2017 May 3;8:230. doi: 10.3389/fphar.2017.00230 (PMC5414545; doi:10.3389/fphar.2017.00230)
Supplement: Supplementary file 1 [file Presentation_1.pdf]

Supplementary Material

Wu-Tou Decoction in Rheumatoid Arthritis: Integrating Network Pharmacology and In Vivo Pharmacological Evaluation

Qingqing Guo[1], Kang Zheng[2], Danping Fan[1], Yukun Zhao[1,3], Li Li[1], Yanqin Bian[3], Xuemei Qiu[4], Xue Liu[4], Ge Zhang 2, Chaoying Ma[4]*, Xiaojuan He[1,2]* and Aiping Lu[2,3]*

*Correspondence: Chaoying Ma, mcy195888@126.com; Xiaojuan He, hxj19@126.com; Aiping Lu, aipinglu@hkbu.edu.hk

1 Supplementary Figures and Tables

1.1 Supplementary Figures

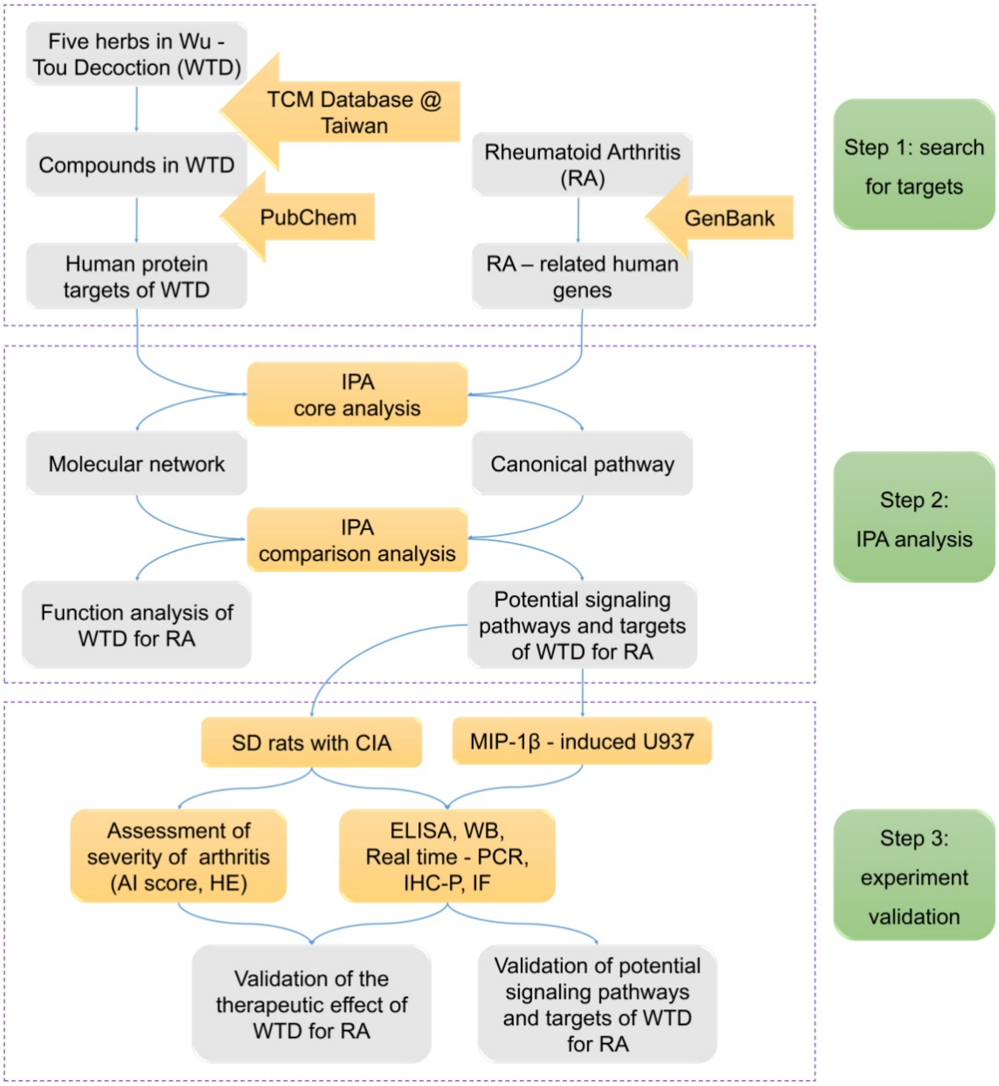

Supplementary Figure 1. Schematic diagram of the network pharmacology-based strategy for exploring action mechanism of WTD in RA. Abbreviations: WTD: Wu-Tou Decoction; TCM: Traditional Chinese Medicine; RA: Rheumatoid Arthritis; IPA: Ingenuity Pathway Analysis; SD: Sprague Dawley; CIA: Collagen Induced Arthritis; MIP-1β: Macrophage Inflammatory Protein-1β; AI: Arthritis Index; HE: Hematoxylin-eosin Staining; ELISA: Enzyme-linked Immunosorbent Assay; WB: Western Blotting; IHC-P: Immunohistochemistry-paraffin section; IF: Immunofluoresence.

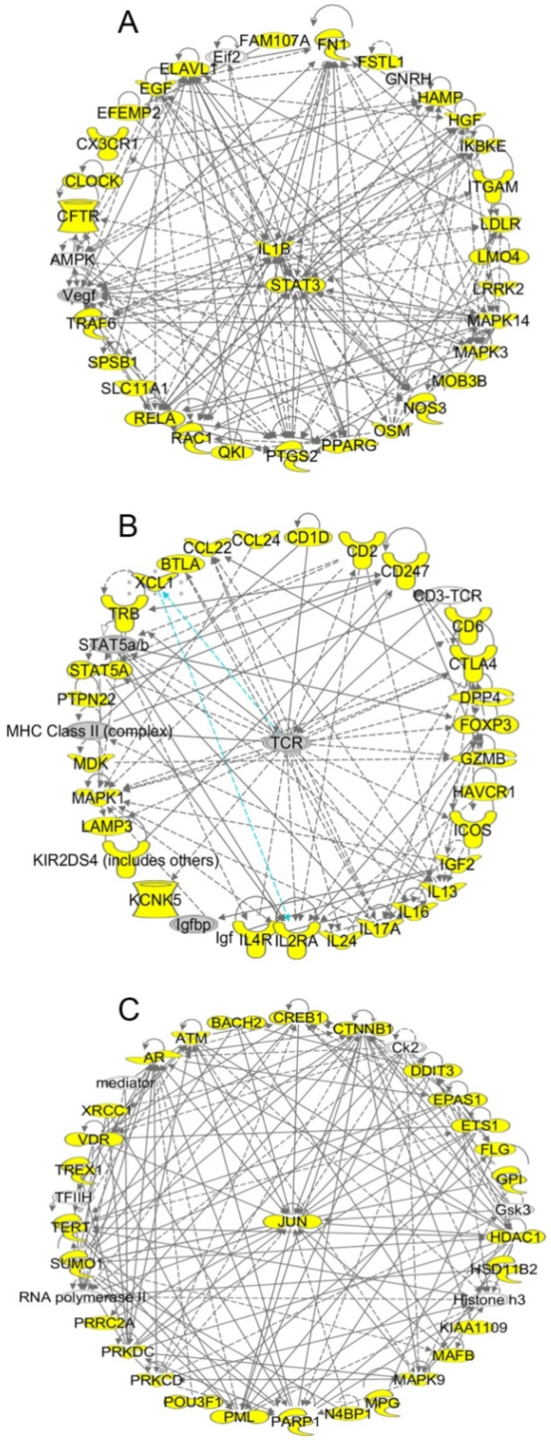

Supplementary Figure 2. Top 3 molecular networks of RA. Yellow nodes represent uploaded molecules and grey nodes represent relevant molecules.

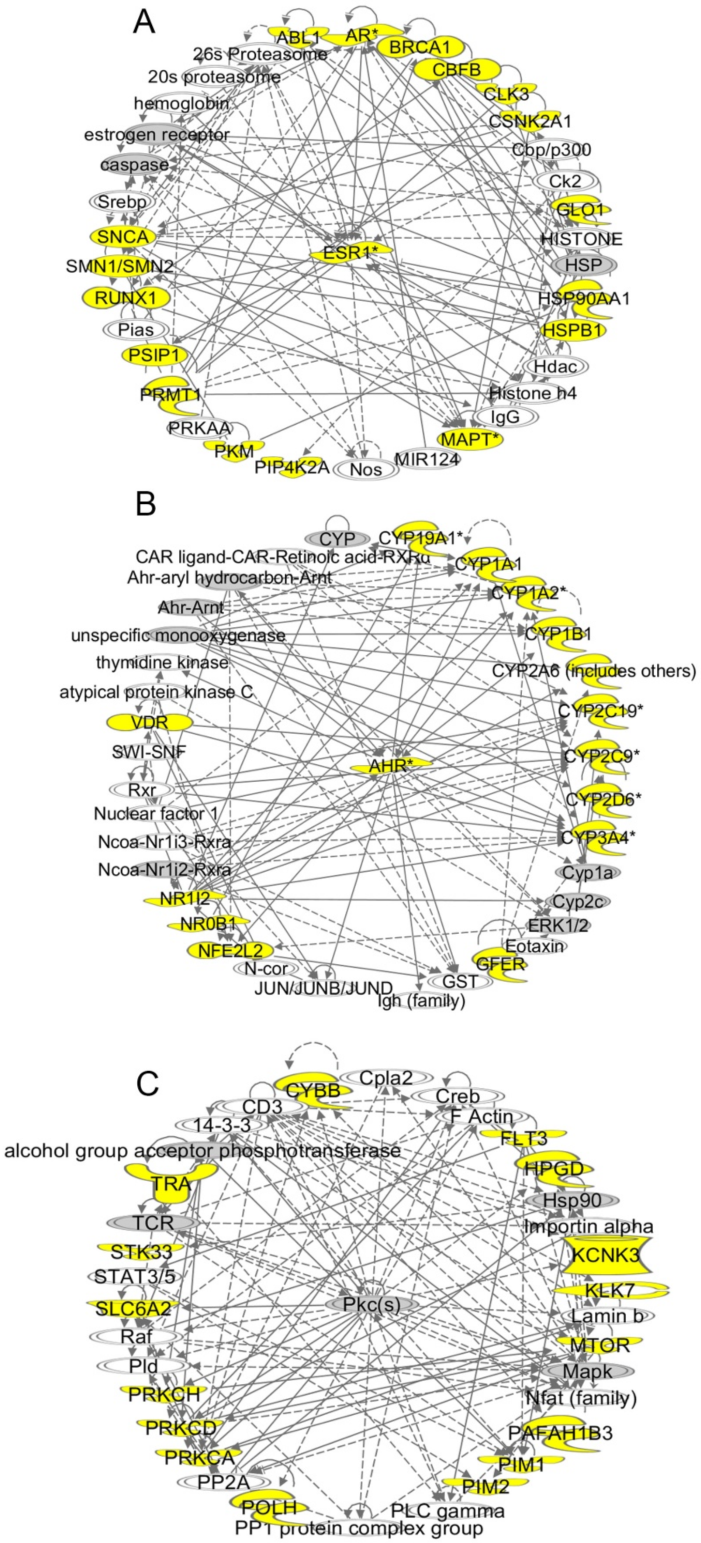

Supplementary Figure 3. Top3 molecular networks of WTD. Yellow nodes represent uploaded molecules and grey nodes represent relevant molecules.

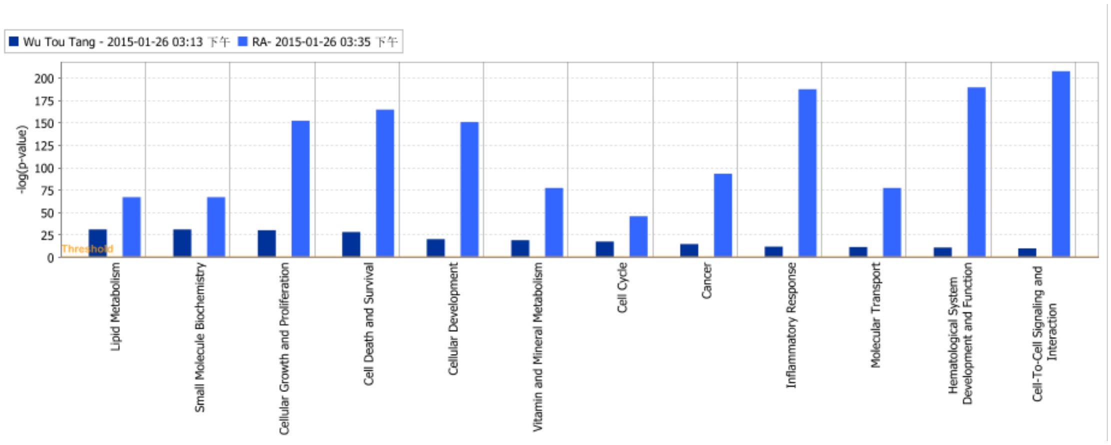

Supplementary Figure 4. Top 12 biofunctions regulated by both targets of WTD and RA-related human genes.

1.2 Supplementary Tables

Supplementary Table1. Chemical compounds of WTD

Herbs in WTD	Chemical Compounds
Aconiti Radix	uracil
Aconiti Radix	songorine
Aconiti Radix	carmichaeline
Aconiti Radix	aconitine
Aconiti Radix	talatisamine
Aconiti Radix	higenamine
Aconiti Radix	aconitan a, b, c, d
Aconiti Radix	mesaconitine
Ephedrae Herba	(-)-Epiafzelechin
Ephedrae Herba	(4S,5R) Ephedroxane
Ephedrae Herba	(4S,5R)Ephedroxane
Ephedrae Herba	1,1,3-Trimethylcyclopentane
Ephedrae Herba	1,3,4-Trimethyl-3-cyclohexene-1-carboxaldehyde
Ephedrae Herba	11-Methoxyhumantenine
Ephedrae Herba	16-Triacontanol
Ephedrae Herba	2,3,4-Trimethyl-5-phenyloxazolidine
Ephedrae Herba	3,4-Dimethyl-5-phenyloxazolidine
Ephedrae Herba	3-Methoxyherbacetin
Ephedrae Herba	3-O-β-D-Glucopyranosyl-5,9,4'-trihydroxy-8-methoxyflavone
Ephedrae Herba	4,5',8-Trimethyl psoralen
Ephedrae Herba	7-Demethylsuberosin
Ephedrae Herba	alpha-Teripineol
Ephedrae Herba	Apigenin
Ephedrae Herba	Apigenin-5-rhamnoside
Ephedrae Herba	Chuanxiongzine
Ephedrae Herba	Cibarian
Ephedrae Herba	cis-p-2-Menthen-1-ol
Ephedrae Herba	D-N-Methyl-pseudoephedrine
Ephedrae Herba	d-Pseudoephedrine
Ephedrae Herba	delta-Terpineol
Ephedrae Herba	Dimethyl phthalate
Ephedrae Herba	Ephedrine
Ephedrae Herba	Glucoraphanin
Ephedrae Herba	Herbacetin
Ephedrae Herba	Herbacetin-3-beta-D-(2-O-beta-D-glucopyra-nosidogluco-pyranoside)-8-beta-D-glucopyranoside
Ephedrae Herba	Kaempferol
Ephedrae Herba	Kaempferol-3-arabofuranoside
Ephedrae Herba	Kaempferol-7-rhamnoside
Ephedrae Herba	Leucodelphinidin
Ephedrae Herba	Leucopelargonidin

Ephedrae Herba	Maokonine
Ephedrae Herba	Maragenin II
Ephedrae Herba	Methyl-7-epiganoderate
Ephedrae Herba	Myrcene
Ephedrae Herba	Myricadiol
Ephedrae Herba	N-Methylephedrine
Ephedrae Herba	n-Triacontanol
Ephedrae Herba	Nor-rubrofusarin
Ephedrae Herba	norephedrine
Ephedrae Herba	Norerythrostachaldine
Ephedrae Herba	O-Methylptelefolonium
Ephedrae Herba	Phenethylcaffeate
Ephedrae Herba	Phenethylamine
Ephedrae Herba	Pseudoginsenoside F11
Ephedrae Herba	trans-belta-Terpineol
Astragali Radix	2-Hydroxy-3-methoxystrychnine
Astragali Radix	20(R)-21,24-Cyclo-3beta,25-dihydroxyl-dammar-23(24)-en-21-one
Astragali Radix	20-Hexadecanoylingenol
Astragali Radix	3,5-Dimethoxystilbene
Astragali Radix	3-O-beta-D-Glucuronopyranosyl gypsogenin
Astragali Radix	4-Hydroxy-2,6-dimethyl-6-(3,7-dimethyl-2,6-octadienyl)-8-(3-methyl-2-butenyl)-2H-1-benzopyran-5,7(3H,6H)-dione
Astragali Radix	Astragaloside I
Astragali Radix	Astragaloside II
Astragali Radix	Astragaloside III
Astragali Radix	Astragaloside IV
Astragali Radix	Astragaloside V
Astragali Radix	Astragaloside VI
Astragali Radix	Astragaloside VII
Astragali Radix	Astragaloside VIII
Astragali Radix	Betaine
Astragali Radix	Chrysanthemaxanthin
Astragali Radix	Folinic acid
Astragali Radix	Foliosidine
Astragali Radix	Gamma-Sitosterol
Astragali Radix	Kumugansine A
Astragali Radix	N-Candicine
Astragali Radix	Suffruticoside A
Paeoniae Radix Alba	12-Methyl tetradecanoic acid
Paeoniae Radix Alba	4-Phenylbicyclo[2,2,2]octan-1-ol
Paeoniae Radix Alba	Albiflorin
Paeoniae Radix Alba	Benzoic acid
Paeoniae Radix Alba	Eugenitin
Paeoniae Radix Alba	Galuteolin
Paeoniae Radix Alba	Gamma-Sitosterol
Paeoniae Radix Alba	Oxypeucedanin
Paeoniae Radix Alba	Paeoniflorin

Paeoniae Radix Alba	Paeonilactone A
Paeoniae Radix Alba	Paeonin
Paeoniae Radix Alba	Paeonol
Paeoniae Radix Alba	Paeonolide
Paeoniae Radix Alba	Pyrethrin II
Paeoniae Radix Alba	Pyrethrosin
Glycyrrhizae Radix EtRhizoma	(E)-1-[2,4-Dihydroxy-3-(3-methyl-2-butenyl)phenyl]-3-(4-hydroxy-3-[3-methyl-2-butenyl) phenyl]-2-propen-1-one
Glycyrrhizae Radix EtRhizoma	2,5-Dihydroxymethyl-3,4-dihydroxypyrrolidine
Glycyrrhizae Radix EtRhizoma	2-Methyl-1,3,6-trihydroxyanthraquinone
Glycyrrhizae Radix EtRhizoma	3,4-Dicaffeoyl-5-(3-hydroxy-3-methyl) glutaroylquinic acid
Glycyrrhizae Radix EtRhizoma	3-Hydroxyglabrol (II)
Glycyrrhizae Radix EtRhizoma	8-Methoxy-5-O-glucoside flavone
Glycyrrhizae Radix EtRhizoma	8-Methyl-10-hydroxylycoctonine
Glycyrrhizae Radix EtRhizoma	alpha-Trihydroxycoprostanic acid
Glycyrrhizae Radix EtRhizoma	Dimethyl sebacate
Glycyrrhizae Radix EtRhizoma	Formononetin-7-glucoside
Glycyrrhizae Radix EtRhizoma	Gamma-Sitosterol
Glycyrrhizae Radix EtRhizoma	Gancaonin B
Glycyrrhizae Radix EtRhizoma	Gancaonin C
Glycyrrhizae Radix EtRhizoma	Gancaonin D
Glycyrrhizae Radix EtRhizoma	Gancaonin E
Glycyrrhizae Radix EtRhizoma	Gancaonin F
Glycyrrhizae Radix EtRhizoma	Gancaonin P-3'-methylether
Glycyrrhizae Radix EtRhizoma	Ganoderic acid A
Glycyrrhizae Radix EtRhizoma	Gloeosteretriol
Glycyrrhizae Radix EtRhizoma	Glycyphyllin
Glycyrrhizae Radix EtRhizoma	Glycyrol
Glycyrrhizae Radix EtRhizoma	Glycyroside
Glycyrrhizae Radix EtRhizoma	Glycyrrhetinic acid
Glycyrrhizae Radix EtRhizoma	Glycyrrhetol
Glycyrrhizae Radix EtRhizoma	Glycyrrhisoflavanone
Glycyrrhizae Radix EtRhizoma	Glycyrrhisoflavone
Glycyrrhizae Radix EtRhizoma	Glycyrrhiza-flavonol A
Glycyrrhizae Radix EtRhizoma	Glycyrrhizic acid
Glycyrrhizae Radix EtRhizoma	Glyeurysaponin
Glycyrrhizae Radix EtRhizoma	Glyyunnanprosapogenin D
Glycyrrhizae Radix EtRhizoma	Gmelofuran
Glycyrrhizae Radix EtRhizoma	Hispaglabridin B
Glycyrrhizae Radix EtRhizoma	Hispidulin
Glycyrrhizae Radix EtRhizoma	Isogosferol
Glycyrrhizae Radix EtRhizoma	Isoliensinine
Glycyrrhizae Radix EtRhizoma	Isoliquiritin
Glycyrrhizae Radix EtRhizoma	Isolobelanine
Glycyrrhizae Radix EtRhizoma	Isoorientin
Glycyrrhizae Radix EtRhizoma	Isoramanone
Glycyrrhizae Radix EtRhizoma	Isotrilobine

Glycyrrhizae Radix EtRhizoma	Lensinine
Glycyrrhizae Radix EtRhizoma	Licochalcone A
Glycyrrhizae Radix EtRhizoma	Licoflavone
Glycyrrhizae Radix EtRhizoma	Licoisoflavaone
Glycyrrhizae Radix EtRhizoma	Licopyranocoumarin
Glycyrrhizae Radix EtRhizoma	Licoricesaponine A3
Glycyrrhizae Radix EtRhizoma	Licoricesaponine C2
Glycyrrhizae Radix EtRhizoma	Licoricesaponine D3
Glycyrrhizae Radix EtRhizoma	Licoricesaponine F3
Glycyrrhizae Radix EtRhizoma	Licoricesaponine G2
Glycyrrhizae Radix EtRhizoma	Licoricesaponine H2
Glycyrrhizae Radix EtRhizoma	Licoricesaponine J2
Glycyrrhizae Radix EtRhizoma	Licoricesaponine K2
Glycyrrhizae Radix EtRhizoma	Licoricidin
Glycyrrhizae Radix EtRhizoma	Licoricone
Glycyrrhizae Radix EtRhizoma	Liquiritigenin-7-O-beta-D-(3-O-acetyl)-apiofuranosyl-4'-O-beta-D-glucopyranoside
Glycyrrhizae Radix EtRhizoma	Liquiritin
Glycyrrhizae Radix EtRhizoma	Liquoric acid
Glycyrrhizae Radix EtRhizoma	Methyl 2-hydroxy-3,4-dimethoxy benzoate
Glycyrrhizae Radix EtRhizoma	Methyl 3-O-beta-D-glucopyranosyl polygalacate
Glycyrrhizae Radix EtRhizoma	Methyl linoleate
Glycyrrhizae Radix EtRhizoma	Methyl-24-hydroxyglycyrrhetate
Glycyrrhizae Radix EtRhizoma	Methylglycyrrhetate
Glycyrrhizae Radix EtRhizoma	Methylglyoxal
Glycyrrhizae Radix EtRhizoma	Narwedine
Glycyrrhizae Radix EtRhizoma	Neohancoside A
Glycyrrhizae Radix EtRhizoma	Neoisopulegol
Glycyrrhizae Radix EtRhizoma	Neomatatabiol
Glycyrrhizae Radix EtRhizoma	Neowilforine
Glycyrrhizae Radix EtRhizoma	Ononitol
Glycyrrhizae Radix EtRhizoma	Phebalosin
Glycyrrhizae Radix EtRhizoma	Ruvoside
Glycyrrhizae Radix EtRhizoma	Tetrahydroharmine
Glycyrrhizae Radix EtRhizoma	Tetrahydropalmatine
Glycyrrhizae Radix EtRhizoma	Uralenin
Glycyrrhizae Radix EtRhizoma	Uralenneoside
Glycyrrhizae Radix EtRhizoma	Uralenol
Glycyrrhizae Radix EtRhizoma	Uralenol-3-methylether
Glycyrrhizae Radix EtRhizoma	Uralsaponin A
Glycyrrhizae Radix EtRhizoma	Uralsaponin B
Glycyrrhizae Radix EtRhizoma	Urea
Glycyrrhizae Radix EtRhizoma	Vicianin

Supplementary Table 2. Human target proteins of WTD

Chemical Compounds	Target Proteins	ID
higenamine	D(2) dopamine receptor	118206
Apigenin	Estradiol 17-beta-dehydrogenase 1	313104233
Apigenin	Delta-type opioid receptor	311033488
Apigenin	ATPase family AAA domain-containing protein 5	296439460
Apigenin	Cellular tumor antigen p53	269849759
Apigenin	Tankyrase-1	226693566
Apigenin	Receptor-type tyrosine-protein kinase FLT3	156630887
Apigenin	Kappa-type opioid receptor	116242691
Apigenin	Casein kinase II subunit alpha	55977123
Apigenin	Cytochrome P450 1B1	48429256
Apigenin	Glycogen synthase kinase-3 beta	20455502
Apigenin	Lysine--tRNA ligase	20178333
Apigenin	Tankyrase-2	20140805
Apigenin	Cyclin-dependent kinase 6	266423
Apigenin	Glucocorticoid receptor	121069
Apigenin	Aromatase	117293
Apigenin	Cytochrome P450 1A2	117144
Apigenin	Cytochrome P450 1A1	117139
Apigenin	Cholinesterase	116353
Apigenin	Cytochrome b-245 heavy chain	115211
Apigenin	Androgen receptor	113830
Apigenin	Acetylcholinesterase	113037
Apigenin	Dual specificity protein kinase CLK1	206729857
Apigenin	Mu-type opioid receptor	2851402
Apigenin	Beta-secretase 1	296434407
Apigenin	AR protein	124375976
Apigenin	SUMO1/sentrin specific peptidase 7	120538355
Apigenin	Microtubule-associated protein tau	92096784
Apigenin	TDP1 protein	79154014
Apigenin	cytochrome P450 2D6 isoform 2	392513723
Apigenin	aldo-keto reductase family 1 member C4	325652083
Apigenin	sentrin-specific protease 8	262118306
Apigenin	dual specificity protein kinase CLK3 isoform b	153791372
Apigenin	cytochrome P450 1A2	73915100
Apigenin	serine/threonine-protein kinase 16	57165436
Apigenin	neuropeptide S receptor isoform A	46395496
Apigenin	serine/threonine-protein kinase pim-2	42821112
Apigenin	guanine nucleotide-binding protein G(i) subunit alpha-1 isoform 1	33946324
Apigenin	15-hydroxyprostaglandin dehydrogenase [NAD(+)] isoform 1	31542939
Apigenin	glycogen synthase kinase-3 beta isoform 1	21361340
Apigenin	nuclear factor erythroid 2-related factor 2 isoform 1	20149576
Apigenin	cytochrome P450 2C9 precursor	13699818
Apigenin	cytochrome P450 3A4 isoform 1	13435386

Apigenin	interleukin-1 beta proprotein	10835145
Apigenin	DNA polymerase kappa	7705344
Apigenin	nuclear receptor subfamily 0 group B member 1	5016090
Apigenin	GTP-binding protein GEM	4885263
Apigenin	serine/threonine-protein kinase pim-1 isoform 2	4505811
Apigenin	cytochrome P450 2C19 precursor	4503219
Apigenin	survival motor neuron protein isoform d	10937869
Apigenin	Chain A, Crystal Structure Of The Human 2-Oxoglutarate Oxygenase Loc390245	221046486
Apigenin	Chain B, The Structure Of Wild-Type Human Hadh2 (17beta-Hydroxysteroid Dehydrogenase Type 10) Bound To Nad+ At 1.2 A	122921311
Apigenin	Chain A, The Structure Of Wild-Type Human Hadh2 (17beta-Hydroxysteroid Dehydrogenase Type 10) Bound To Nad+ At 1.2 A	122921310
Apigenin	Chain A, Human Ape1 Endonuclease With Bound AbasicDna And Mn2+ Ion	6980812
Apigenin	aryl hydrocarbon receptor	51095037
Apigenin	estrogen nuclear receptor alpha	348019627
Apigenin	Parkin	3063388
Apigenin	pregnane X nuclear receptor	325495557
Apigenin	farnesoid X nuclear receptor	325495553
Apigenin	glucocorticoid receptor	311348376
Apigenin	SUMO-1-specific protease	616648
Apigenin	RGS12	3290016
Apigenin	BRCA1	1698399
Apigenin	hypoxia-inducible factor 1, alpha subunit (basic helix-loop-helix transcription factor)	32879895
Apigenin	aldehyde dehydrogenase 1 family, member A1	30582681
Apigenin	15-lipoxygenase, partial	1832253
Apigenin	vitamin D (1,25- dihydroxyvitamin D3) receptor	216409708
Apigenin	peroxisome proliferator activated receptor gamma	216409692
Apigenin	peroxisome proliferator-activated receptor delta	216409690
d-Pseudoephedrine	Arachidonate 15-lipoxygenase B	317373425
d-Pseudoephedrine	Thrombopoietin	120660324
d-Pseudoephedrine	cytochrome P450 2D6 isoform 1	392513721
d-Pseudoephedrine	cytochrome P450 1A2	73915100
d-Pseudoephedrine	guanine nucleotide-binding protein G(i) subunit alpha-1 isoform 1	33946324
d-Pseudoephedrine	pyruvate kinase PKM isoform b	33286420
d-Pseudoephedrine	cytochrome P450 2C9 precursor	13699818
d-Pseudoephedrine	cytochrome P450 3A4 isoform 1	13435386
d-Pseudoephedrine	cytochrome P450 2C19 precursor	4503219
d-Pseudoephedrine	Chain B, The Structure Of Wild-Type Human Hadh2 (17beta-Hydroxysteroid Dehydrogenase Type 10) Bound To Nad+ At 1.2 A	122921311
d-Pseudoephedrine	Chain A, The Structure Of Wild-Type Human Hadh2 (17beta-Hydroxysteroid Dehydrogenase	122921310

	Type 10) Bound To Nad+ At 1.2 A	
d-Pseudoephedrine	RGS12	122921310
d-Pseudoephedrine	15-lipoxygenase, partial	1832253
d-Pseudoephedrine	alpha-galactosidase	757912
Kaempferol	Estradiol 17-beta-dehydrogenase	313104233
Kaempferol	Steroid hormone receptor ERR1	215274146
Kaempferol	Receptor-type tyrosine-protein kinase FLT3	156630887
Kaempferol	Lactoylglutathionelyase	134039205
Kaempferol	Cytochrome P450 1B1	48429256
Kaempferol	Phosphatidylinositol 5-phosphate 4-kinase type-2 alpha	18266879
Kaempferol	Estradiol 17-beta-dehydrogenase 2	544152
Kaempferol	Cyclin-dependent kinase 6	266423
Kaempferol	Arachidonate 5-lipoxygenase	126407
Kaempferol	Cytochrome P450 1A2	117144
Kaempferol	Cytochrome P450 1A1	117139
Kaempferol	Cholinesterase	116353
Kaempferol	Androgen receptor	113830
Kaempferol	Aldose reductase	113596
Kaempferol	disintegrin and metalloproteinase domain-containing protein 17 preproprotein	73747889
Kaempferol	Dual specificity protein kinase CLK1	206729857
Kaempferol	Sialate O-acetylesterase	74734243
Kaempferol	Beta-secretase 1	296434407
Kaempferol	racGTPase-activating protein 1	21361397
Kaempferol	AR protein	124375976
Kaempferol	Microtubule-associated protein tau	92096784
Kaempferol	cytochrome P450 2D6 isoform 2	392513723
Kaempferol	cytochrome P450 2D6 isoform 1	392513721
Kaempferol	platelet-activating factor acetylhydrolase IB subunit gamma	225543099
Kaempferol	DNA polymerase iota	154350220
Kaempferol	cytochrome P450 1A2	73915100
Kaempferol	serine/threonine-protein kinase 16	57165436
Kaempferol	serine/threonine-protein kinase pim-2	42821112
Kaempferol	muscarinic acetylcholine receptor M1	37622910
Kaempferol	15-hydroxyprostaglandin dehydrogenase [NAD(+)] isoform 1	31542939
Kaempferol	glycogen synthase kinase-3 beta isoform 1	21361340
Kaempferol	nuclear factor erythroid 2-related factor 2 isoform 1	20149576
Kaempferol	cytochrome P450 2C9 precursor	13699818
Kaempferol	cytochrome P450 3A4 isoform 1	13435386
Kaempferol	carboxy-terminal domain RNA polymerase II polypeptide A small phosphatase 1 isoform 1	10864009
Kaempferol	DNA polymerase eta isoform 1	5729982
Kaempferol	cytochrome P450 2C19 precursor	4503219
Kaempferol	cytochrome P450, family 19, subfamily A,	119597822

	polypeptide 1, isoform CRA_a	
Kaempferol	Chain B, The Structure Of Wild-Type Human Hadh2 (17beta-Hydroxysteroid Dehydrogenase Type 10) Bound To Nad+ At 1.2 A	122921311
Kaempferol	Chain A, The Structure Of Wild-Type Human Hadh2 (17beta-Hydroxysteroid Dehydrogenase Type 10) Bound To Nad+ At 1.2 A	122921310
Kaempferol	aryl hydrocarbon receptor	51095037
Kaempferol	estrogen nuclear receptor alpha	348019627
Kaempferol	pregnane X nuclear receptor	325495557
Kaempferol	glucocorticoid receptor	311348376
Kaempferol	ADAM10	2393947
Kaempferol	hypoxia-inducible factor 1, alpha subunit (basic helix-loop-helix transcription factor)	32879895
Kaempferol	peroxisome proliferator activated receptor gamma	216409692
Myrcene	heat shock protein beta-1	4504517
Phenethylcaffeate	Aldo-keto reductase family 1 member B10	322510010
Phenethylcaffeate	Arachidonate 15-lipoxygenase B	317373425
Phenethylcaffeate	Prostaglandin G/H synthase 1	317373262
Phenethylcaffeate	Aldo-keto reductase family 1 member C3	308153646
Phenethylcaffeate	Aldo-keto reductase family 1 member C4	308153631
Phenethylcaffeate	TAR DNA-binding protein 43	20140568
Phenethylcaffeate	Epidermal growth factor receptor	2811086
Phenethylcaffeate	Alpha-synuclein	586067
Phenethylcaffeate	Estrogen receptor	544257
Phenethylcaffeate	Aldo-keto reductase family 1 member C1	416877
Phenethylcaffeate	Sodium-dependent noradrenaline transporter	128616
Phenethylcaffeate	Tyrosine-protein kinase Lck	125474
Phenethylcaffeate	Tyrosine-protein kinase Fyn	125370
Phenethylcaffeate	Mitogen-activated protein kinase 14	2499600
Phenethylcaffeate	Receptor tyrosine-protein kinase erbB-2	119533
Phenethylcaffeate	Aldose reductase	113596
Phenethylcaffeate	Aldo-keto reductase family 1 member C2	20532374
Phenethylcaffeate	ATAD5 protein, partial	116283940
Phenethylcaffeate	TDP1 protein	79154014
Phenethylcaffeate	platelet-activating factor acetylhydrolase IB subunit beta isoform b	296080766
Phenethylcaffeate	heat shock protein HSP 90-alpha isoform 2	154146191
Phenethylcaffeate	serine/threonine-protein kinase mTOR	4826730
Phenethylcaffeate	FAD-linked sulfhydryl oxidase ALR	54112432
Phenethylcaffeate	survival motor neuron protein isoform d	10937869
Phenethylcaffeate	Chain A, Crystal Structure Of The Human 2-Oxoglutarate Oxygenase Loc390245	221046486
Phenethylcaffeate	Chain B, The Structure Of Wild-Type Human Hadh2 (17beta-Hydroxysteroid Dehydrogenase Type 10) Bound To Nad+ At 1.2 A	122921311
Phenethylcaffeate	Chain A, The Structure Of Wild-Type Human Hadh2 (17beta-Hydroxysteroid Dehydrogenase	122921310

	Type 10) Bound To Nad+ At 1.2 A	
Phenethylcaffeate	Chain A, Jmjd2a Tandem Tudor Domains In Complex With A Trimethylated Histone H4-K20 Peptide	162330054
Phenethylcaffeate	GLS protein	71051501
Phenethylcaffeate	90-kda heat shock protein beta HSP90 beta, partial	341916350
Phenethylcaffeate	Kcnk3 channel	11093520
Phenethylcaffeate	15-lipoxygenase, partial	1832253
Phenethylcaffeate	serine/threonine kinase 33	12830367
Phenethylamine	Cytochrome P450 2A6	308153612
Phenethylamine	Trace amine-associated receptor 1	38258636
Phenethylamine	trace amine-associated receptor 1	21264324
Betaine	alkaline phosphatase, tissue-nonspecific isozyme isoform 1 precursor	116734717
Betaine	protein-arginine deiminase type-4	216548487
Betaine	racGTPase-activating protein 1	21361397
Betaine	kallikrein-7 isoform 1 preproprotein	21327705
Betaine	high affinity choline transporter 1	11141885
Betaine	alkaline phosphatase, placental-like preproprotein	157266296
Betaine	Chain A, Structure Of Human Recq-Like Helicase In Complex With A Dna Substrate	282403581
Betaine	Chain A, Human Ape1 Endonuclease With Bound AbasicDna And Mn^{2+} Ion	6980812
Betaine	PSMD14 protein	16306916
Betaine	PRMT1 protein	32425330
Folinic acid	Folate transporter 1	12643280
Benzoic acid	D-amino-acid oxidase	25453448
Benzoic acid	estrogen nuclear receptor alpha	348019627
Oxypeucedanin	Chain A, Crystal Structure Of The Human 2-Oxoglutarate Oxygenase Loc390245	221046486
Oxypeucedanin	TDP1 protein	79154014
Oxypeucedanin	TTR	48145933
Oxypeucedanin	aryl hydrocarbon receptor precursor	4502003
Paeonol	15-hydroxyprostaglandin dehydrogenase [NAD(+)] isoform 1	31542939
Formononetin-7-glucoside	ATAD5 protein, partial	116283940
Formononetin-7-glucoside	Niemann-Pick C1 protein precursor	255652944
Formononetin-7-glucoside	ras-related protein Rab-9A	4759012
Glycyrrhetinic acid	Aldo-keto reductase family 1 member B10	322510010
Glycyrrhetinic acid	Protein kinase C alpha type	317373571
Glycyrrhetinic acid	Protein kinase C eta type	281185512
Glycyrrhetinic acid	Protein kinase C delta type	205371776
Glycyrrhetinic acid	Cytochrome P450 3A4	116241312
Glycyrrhetinic acid	Cytochrome P450 2D6	84028191
Glycyrrhetinic acid	Cytochrome P450 2C19	60416369
Glycyrrhetinic acid	Corticosteroid 11-beta-dehydrogenase isozyme 2	30316367
Glycyrrhetinic acid	Protein kinase C beta type	20141488

Glycyrrhetinic acid	Peroxisome proliferator-activated receptor gamma	13432234
Glycyrrhetinic acid	Cytochrome P450 2C9	6686268
Glycyrrhetinic acid	Corticosteroid 11-beta-dehydrogenase isozyme 1	118569
Glycyrrhetinic acid	TDP1 protein	79154014
Glycyrrhetinic acid	aldo-keto reductase family 1 member C4	325652083
Glycyrrhetinic acid	DNA polymerase kappa	7705344
Glycyrrhetinic acid	corticosteroid 11-beta-dehydrogenase isozyme 1	5031765
Glycyrrhetinic acid	heat shock protein beta-1	4504517
Glycyrrhetinic acid	glucocorticoid receptor	311348376
Glycyrrhizic acid	Corticosteroid 11-beta-dehydrogenase isozyme 2	30316367
Glycyrrhizic acid	Corticosteroid 11-beta-dehydrogenase isozyme 1	118569
Glycyrrhizic acid	AR protein	124375976
Glycyrrhizic acid	glucocorticoid receptor	311348376
Hispidulin	SUMO1/sentrin specific peptidase 7	120538355
Hispidulin	ATAD5 protein, partial	116283940
Hispidulin	TDP1 protein	79154014
Hispidulin	sentrin-specific protease 8	262118306
Hispidulin	nuclear receptor subfamily 0 group B member 1	5016090
Hispidulin	FAD-linked sulfhydryl oxidase ALR	54112432
Hispidulin	caspase-3 preproprotein	14790119
Hispidulin	SUMO-1-specific protease	6166485
Isoliquiritin	Aldose reductase	113596
Isoorientin	Aldose reductase	113596
Isoorientin	racGTPase-activating protein 1	21361397
Isoorientin	DNA polymerase iota	154350220
Isoorientin	ras and Rabinteractor 1	68989256
Isoorientin	tyrosine-protein kinase ABL1 isoform a	62362414
Isoorientin	DNA polymerase kappa	7705344
Isoorientin	FAD-linked sulfhydryl oxidase ALR	54112432
Isoorientin	Chain A, Jmjd2a Tandem Tudor Domains In Complex With A Trimethylated Histone H4-K20 Peptide	162330054
Isoorientin	lens epithelium-derived growth factor p75	6708281
Licochalcone A	Tyrosine-protein phosphatase non-receptor type 1	131467
Licoflavone	cGMP-specific 3',5'-cyclic phosphodiesterase	317373261
Licoflavone	serine/threonine kinase 33	12830367
Licoflavone	Peroxisome proliferator-activated receptor gamma	13432234
Liquiritin	Prolyl 4-hydroxylase, beta polypeptide	14790033
Liquoric acid	Aldose reductase	113596
Methyl linoleate	AR protein	124375976
Methyl linoleate	heat shock protein beta-1	4504517
Methyl linoleate	glucocorticoid receptor	311348376
Tetrahydroharmine	Prolylendopeptidase-like	153217451
Tetrahydroharmine	TDP1 protein	79154014
Tetrahydroharmine	protein AF-9 isoform a	156104889
Tetrahydroharmine	runt-related transcription factor 1 isoform AML1c	19923198
Tetrahydroharmine	core-binding factor subunit beta isoform 1	13124881
Tetrahydroharmine	mitogen-activated protein kinase	22035600

	kinasekinasekinase 2	
Tetrahydroharmine	Chain A, Jmjd2a Tandem Tudor Domains In Complex With A Trimethylated Histone H4-K20 Peptide	162330054
Tetrahydroharmine	PSMD14 protein	16306916
Tetrahydroharmine	TCRAV4S1, partial	2358024
Tetrahydropalmatine	Tissue factor	135666
Tetrahydropalmatine	D(1A) dopamine receptor	118228

Supplementary Table 3. Molecular networks of RA

ID	Molecules in Network	Score	Top Diseases and Functions
1	AMPK, CFTR, CLOCK, CX3CR1, EFEMP2, EGF, Eif2, ELAVL1, FAM107A, FN1, FSTL1, GNRH, HAMP, HGF, IKBKE, IL1B, ITGAM, LDLR, LMO4, LRRK2, MAPK3, MAPK14, MOB3B, NOS3, OSM, PPARG, PTGS2, QKI, RAC1, RELA, SLC11A1, SPSB1, STAT3, TRAF6, Vegf	40	Cellular Movement, Hematological System Development and Function, Immune Cell Trafficking
2	BTLA, CCL22, CCL24, CD2, CD6, CD247, CD1D, CD3-TCR, CTLA4, DPP4, FOXP3, GZMB, HAVCR1, ICOS, Igf, IGF2, Igfbp, IL13, IL16, IL24, IL17A, IL2RA, IL4R, KCNK5, KIR2DS4 (includes others), LAMP3, MAPK1, MDK, MHC Class II (complex), PTPN22, STAT5A, STAT5a/b, TCR, TRB, XCL1	40	Cell-To-Cell Signaling and Interaction, Hematological System Development and Function, Immune Cell Trafficking
3	AR, ATM, BACH2, Ck2, CREB1, CTNNB1, DDIT3, EPAS1, ETS1, FLG, GPI, Gsk3, HDAC1, Histone h3, HSD11B2, JUN, KIAA1109, MAFB, MAPK9, mediator, MPG, N4BP1, PARP1, PML, POU3F1, PRKCD, PRKDC, PRRC2A, RNA polymerase II, SUMO1, TERT, TFIIH, TREX1, VDR, XRCC1	36	Cell Death and Survival, Gene Expression, Cell Cycle
4	26s Proteasome, ADIPOR1, BCL2, CASC3, CD44, CDK4, CDK6, CDKN1A, CSF1, CUL1, Cyclin D1/cdk4, DHFR, DNMT3B, ERBB2, FKBP5, FOXO3, HIF1A, HNRNPA2B1, Hsp90, MBD4, MDM2, MITF, MMP1, MTHFR, MYC, NGFR, NLRP1, NOS2, NR3C1, RB1, RUNX1, TNFRSF1B, TNPO3, TXNDC5, UBE2L3	36	Cell Cycle, Cancer, Tumor Morphology
5	ADORA2A, C5, C5AR1, CCL5, CCR1, CCR2, CCR5, CCR9, CD28, CD84, CD276, chemokine, CNR2, CSF2, CXCL8, CXCL16, G-protein beta, G-protein gamma, GCH1, IFNG, IL15, IL12 (family), IL6R, Interferon alpha, KIR2DL1/KIR2DL3, MERTK, mir-15, P110, PTGES, Shc, TNFRSF9, TNFSF15, TREM1, U1 snRNP, ZFP36	34	Inflammatory Response, Cellular Movement, Hematological System Development and Function
6	ADAMTS4, ADAMTS5, Ap1, ARAP2, ARID5B, BCR (complex), CARD8, CCL3, CD40, CD40LG, CTSB, CX3CL1, Egfr-Erbb2, FAS, FASLG, FCAR, GFAP, Gi-coupled receptor, Growth hormone, HMGB1, HOXD9, Igm, IL1, IL10, IL1RN, IRF1, KPNA1, LST1, Mmp, NLRP3, P2RX7, PFKFB3, PRDM1, PSTPIP1, Sapk	32	Connective Tissue Disorders, Inflammatory Disease, Skeletal and Muscular Disorders
7	AIRE, BCL6, BCL2L1, BID, BTK, calpain, CD3, CD4, CFLAR, DDX6, DDX39B, ENO1, FCRL3, HMOX1, IL2, IL21R, ITCH, ITGA4, ITGB2, MAPK8, MBP, MSR1, MSRA, MTOR, p70 S6k,	31	Cell Death and Survival, Cellular Development, Cellular Growth and Proliferation

	PIN1, PRKCB, PRKCZ, RAF1, Rap1, SH2B3, SYK, TIA1, TSH, VCAM1		
8	A2M, ADIPOQ, Alp, APOB, APOE, BST1, C3, CHRNA7, CLU, F3, HSP, Hsp27, Hsp70, HSP90B1, HSPA5, HSPB8, HSPD1, IFN Beta, IL12B, LEP, LRP1, MMP3, MMP14, MT-CO2, NADPH oxidase, NOX1, NR4A2, P38 MAPK, PDGF BB, Plc beta, Pro-inflammatory Cytokine, SELP, SERCA, SERPINA1, SUMO4	28	Cardiovascular System Development and Function, Cellular Movement, Cellular Development
9	C8, C-C chemokine receptor, CCL2, CCL13, CCL18, CCL3L1, chitinase, CXCL10, CXCL13, Icam, IFNK, IL33, IL-1R/TLR, IRF5, LGALS2, lipoxygenase, LTA, LTB, LTBR, Lymphotoxin, Mac, mir-146, NfkB-RelA, PRTN3, SIGIRR, TIMD4, TLR5, TLR8, TLR9, TLR10, Tnf receptor, TNFRSF18, TNFSF14, TRAF2-TRAF3, tryptase	27	Cell-To-Cell Signaling and Interaction, Hematological System Development and Function, Immune Cell Trafficking
10	Calmodulin, Caveolin, CD14, CD38, Ikb, IL1A, IRAK1, IRAK1/4, NFKB1, NFkB1-CRel, NFKBIA, NFKBIE, NOD1, NOD2, PFKL, PLA2, polyubiquitin, REL, RelA-CRel, SELE, SPA17, SPHK2, TAB2, TAB2/TAB3, TIRAP, TLR2, TLR4, TNFAIP3, TNFRSF10B, TNFRSF1A, TRP, TRPC1, TRPC5, Ube3, Ubiquitin	26	Cell-To-Cell Signaling and Interaction, Hematological System Development and Function, Immune Cell Trafficking
11	ALPL, BMP4, CALCA, Collagen type V, Collagen Type XI, CTGF, CXCR4, DHCR7, EDN1, Enolase, ESR2, estrogen receptor, FSH, Gαq, HPSE, IGF1, Itgam-Itgb2, ITGB1, JAM3, Lh, MMP2, MMP9, NEDD9, PADI2, PLAU, PRL, PTPN2, Rab5, SALL3, SERPINE1, SRC (family), Tgf beta, THBS1, tyrosine kinase, VIM	26	Cellular Movement, Cancer, Tumor Morphology
12	ANGPT1, ANGPT2, BAK1, BIRC3, BMX, BSG, c-Src, CD274, CSF1R, CXCR3, Dynamin, EFNB1, ENaC, Fgf, Fgfr, Gap, I kappa b kinase, IL34, IL7R, Il8r, NFkB (family), PARP, phosphatidylinositol-4,5-bisphosphate 3-kinase, PI3K (family), PI3K p85, PIK3CA, PIK3CD, PROC, PTGS1, PTK2, SDC3, TEK, TNFSF10, TNIP1, TYRO3	26	Hematological System Development and Function, Tissue Morphology, Inflammatory Response
13	Anti-inflammatory Cytokine, arginase, Arnt-Hif1a, ATP1A1, CD69, Cpla2, FCN1, GUSB, HAS1, HAS2, IL4, Il-4 receptor, KLRC4-KLRK1/KLRK1, Ku, LDL, MEFV, MMR, MTORC2, MTRR, NAMPT, NCR3, Nos, NTN1, OLR1, PLA2G2A, PLA2G4A, sphingomyelinase, TGFB1, TNFAIP6, TPT1, TWIST, UNC5, UNC5B, UNC5C, VTCN1	26	Connective Tissue Disorders, Inflammatory Disease, Skeletal and Muscular Disorders

14	ABCB1, ADM, AHR, Ahr-aryl hydrocarbon-Arnt, ALOX5, APLN, APOA1, ATPase, CAT, Ces, Cyp1a, CYP1A2, cytochrome-c oxidase, Daf, glutathione peroxidase, glutathione transferase, GPX1, GST, GSTM1, GSTP1, GSTT1, ICAM1, IFI16, IKZF3, Ldh (complex), mir-223, MUC1, Ncoa-Nr1i2-Rxra, PXR ligand-PXR-Retinoic acid-RXRα, RETN, ROBO3, Sod, SOD1, TNFSF9, UGT	24	Free Radical Scavenging, Drug Metabolism, Protein Synthesis
15	CCL23, CD58, CD80, CD83, CD86, CD163, CD209, CLEC4E, CLEC7A, CP, CTNNβ-TCF/LEF, CYP, CYP19, elastase, Fcgr3, FPR, Gm-csf, HLA-DR, Ifn ,Ifn gamma, Ige, IL6, IL18, IL37, IL7R (complex), IRF8, LGALS9, MHC II, mir-155, mir-346, Tlr, TLR3, TLR7, TNFRSF11B, ZC3H12A	24	Cell-To-Cell Signaling and Interaction, Hematological System Development and Function, Immune Cell Trafficking
16	ACKR3, Adaptor protein 2, ADRB3, Beta Arrestin, C-X-C chemokine receptor, CALCRL, CCR3, CCR4, CCR6, CCRL2, chemokine receptor, CHRM3, Clathrin, CMKLR1, EGFR ligand, FPR1, FPR2, FZD5, GHSR, Gpcr, Gs-coupled receptor, HRH4, HTR2A, LPAR3, LTB4R, LTB4R2, LTB4R/LTB4R2, Metalloprotease, MTNR1B, PI3Kγ, Pik3r, RAMP2, Relaxin, Trk Receptor, VIPR1	24	Cell Signaling, Molecular Transport, Vitamin and Mineral Metabolism
17	alcohol group acceptor phosphotransferase, BAD, BCL2L11, BRAF, caspase, Caspase 3/7, Cg, collagen, CST5, CTSS, Cyclin B, E3 RING, EGR1, INHA, INHBA, Inhibin, KLF12, MAP2K4, MAP2K6, Mek, MIF, MS4A1, MYO9B, NPPB, PRKCH, PRKCQ, Raf, Ras homolog, Rsk, S1PR, SH3RF1, SPHK1, TAB2-TAK1, UNG, XIAP	23	Cell Death and Survival, Hematological System Development and Function, Tissue Morphology
18	BPI, CD80/CD86, Collagen type VII, ETFA, GOT, HEXA, HEXB, hexosaminidase, HLA-DMA, HLA-DMB, HLA-DPB1, HLA-DQ, HLA-DQA1, HLA-DQA2, HLA-DQB1, HLA-DQB2, HLA-DRA, Hla-Drb, HLA-DRB1, HLA-DRB4, IL1/IL6/TNF, LILRA3, lymphotoxin-alpha1-beta2, MHC II-β ,Mhc2 Alpha, MIR124, NFKBIL1, prostaglandin-E synthase, Ptger, RARRES2, SCAVENGER receptor CLASS A, SLC22A4, TNF, TNF-A/B, TNFRSF6B	22	Connective Tissue Disorders, Immunological Disease, Inflammatory Disease
19	AICDA, AP-3, apyrase, atypical protein kinase C, Bvr, CENPJ, cyclooxygenase, FADD, Ggt, Ho, Hspg, Iga, Il15r, Mucin, Nfatc, NFkB (complex), PDCD5, PPARα-RXRα, REL/RELA/RELB, TNFRSF8, TNFRSF14, TNFRSF17, TNFRSF25, TNFRSF10A, TNFRSF10D, TNFRSF12A, TNFSF8, TNFSF12, TNFSF13, TNFSF13B, TPMT, TRAF1, TRAF2, UBE2, VIMP	20	Cancer, Cell Death and Survival, Tumor Morphology

20	ACE, ADA, ADAM10, ADAM15, ADAM17, ALOX15, Arf, CHI3L1, DLL1, endothelin receptor, Fibrinogen, Focal adhesion kinase, Integrin, JAG1, Kallikrein, MAP2K1/2, MMP13, N-Cadherin, Neuropilin, Notch, NR2F2, NRP1, NRP2, Pld, PLD4, RBPJ, secreted MMP, Sema3, SEMA3A, SEMA3C, Smad, STAT1/3/5 dimer, Thioredoxin reductase, TPSAB1/TPSB2, VEGFA	20	Cardiovascular System Development and Function, Organismal Development, Embryonic Development
21	15-LOX, ALDH, ALT, ARAP1, ATIC, BANK1, BLK, C4, C1q, C1QB, C1QC, C3-Cfb, C4BP, CFH, Collagen type ix, COMP, Complement component 1, CR1, CST3, DEFA1 (includes others), Dgk, EGFR, ERBB, F3-F7, FAP, FCN3, IgD, MBL2, NRG (family), peptidase, PKC alpha/beta, PXK, Serine Protease, SFTPD, UBASH3A	19	Antimicrobial Response, Humoral Immune Response, Infectious Disease
22	A1BG, AHSG, APOM, C4A/C4B, Camkk, Cebp, CETP, Collagen type II, ERK1/2, Ferritin, GC, GHRL, H19, HDL, HDL-cholesterol, hemoglobin, Hla-abc, HP, IL36A, IL36B, KLRC2, MIR320, MPO, myosin-light-chain kinase, NfkB1-RelA, ORM1, PON1, PRKAA, Rap, SAA, SAA1, Sphk, TF, Trail-R, XDH	19	Lipid Metabolism, Molecular Transport, Small Molecule Biochemistry
23	FLT3LG, FOLR2, FOS, HAVCR2, IFN alpha receptor, IFN alpha/beta, IFNGR1, IL7, IL27, IL10R, IL12 (complex), Il12 receptor, IL23A, IL23R, IL2RB, IL6 receptor, IL6ST, JAK, mir-132, PDCD1, SLC19A1, SOCS, SOCS1, STAT4, Stat dimer, Stat1-Stat3, Stat1/3, STAT4 dimer, TBX21, TH1 Cytokine, TLR2/3/4, TLR3/4, Type 2 Osm Receptor, UBIQUITIN LIGASE, WSX1-gp130	17	Cellular Development, Hematological System Development and Function, Hematopoiesis
24	ARL15, BTNL2, CLEC16A, CTAGE5, CUTC, CYP2E1, CYP4F11, CYP7B1, ENGASE, GGH, GRIPAP1, LECT2, MIA3, MICAL1, MICAL2, NAA20, NAA25, NADSYN1, NBR1, PDE6D, PIP4K2A, PIP4K2C, PIP5K1B, POR, RPL8, RPL9, SEC24C, SLC22A5, SLCO1C1, SPRED2, STEAP4, TFG, UBC, XRN2, YDJC	16	Drug Metabolism, Small Molecule Biochemistry, Molecular Transport
25	AGER, Aldose Reductase, C-type LECTIN, C1QA, CRP, Fc gamma receptor, Fc receptor, Fcgr2, FCGR1A, FCGR2A, FCGR2B, FCGR3A/FCGR3B, Hnp alpha, IgG, IgG1, Igg2, Igg3, IgG4, IL20, IL21, IL22, immune complex, Immunoglobulin, Mac1, Mapk, MIA, S100, S100A4, S100A8, S100A9, S100A12, Syndecan, Tenascin, TH17 Cytokine, TNIP3	16	Cellular Function and Maintenance, Inflammatory Response, Cell-To-Cell Signaling and Interaction

Supplementary Table 4. Molecular networks of WTD

ID	Molecules in Network	Score	Top Diseases and Functions
1	20s proteasome, 26s Proteasome, ABL1, AR, BRCA1, caspase, CBFB, Cbp/p300, Ck2, CLK3, CSNK2A1, ESR1, estrogen receptor, GLO1, Hdac, hemoglobin, HISTONE, Histone h4, HSP, HSP90AA1, HSPB1, IgG, MAPT, MIR124, Nos, Pias, PIP4K2A, PKM, PRKAA, PRMT1, PSIP1, RUNX1, SMN1/SMN2, SNCA, Srebp	30	Cell Death and Survival, Cellular Assembly and Organization, Cancer
2	AHR, Ahr-Arnt, Ahr-aryl hydrocarbon-Arnt, atypical protein kinase C, CAR ligand-CAR-Retinoic acid-RXRα, CYP, CYP19A1, Cyp1a, CYP1A1, CYP1A2, CYP1B1, CYP2A6 (includes others), Cyp2c, CYP2C9, CYP2C19, CYP2D6, CYP3A4, Eotaxin, ERK1/2, GFER, GST, Igh (family), JUN/JUNB/JUND, N-cor, Ncoa-Nr1i2-Rxra, Ncoa-Nr1i3-Rxra, NFE2L2, NR0B1, NR1I2, Nuclear factor 1, Rxr, SWI-SNF, thymidine kinase, unspecific monooxygenase, VDR	26	Lipid Metabolism, Small Molecule Biochemistry, Vitamin and Mineral Metabolism
3	14-3-3, alcohol group acceptor phosphotransferase, CD3, Cpla2, Creb, CYBB, F Actin, FLT3, HPGD, Hsp90, Importin alpha, KCNK3, KLK7, Lamin b, Mapk, MTOR, Nfat (family), PAFAH1B3, PIM1, PIM2, Pkc(s), PLC gamma, Pld, POLH, PP1 protein complex group, PP2A, PRKCA, PRKCD, PRKCH, Raf, SLC6A2, STAT3/5, STK33, TCR, TRA	25	Post-Translational Modification, Cancer, Inflammatory Response
4	Actin, Alpha catenin, CaMKII, CASP3, Cg, ERBB2, ESRRA, FSH, Gamma tubulin, Growth hormone, Gsk3, HIF1A, Histone h3, HSD17B1, HSD17B2, Ifn, IL1B, Lh, MAPK1, Mitochondrial complex 1, NPC1, PDE5A, Pka, PPARD, PTGS1, RACGAP1, RNA polymerase II, RPA, RPL15, SLC19A1, Smad2/3, TCF, TP53, Vegf, VitaminD3-VDR-RXR	25	Cellular Development, Cellular Growth and Proliferation, Energy Production
5	Ahr-aryl hydrocarbon, Ahr-aryl hydrocarbon-RelA, ATAD5, Bcl9-Cbp/p300-Ctnnb1-Lef/Tcf, beta-estradiol, BOP1, CHPT1, DAO, EP300, FADS3, GLA, IFT122, IGK, KIAA0922, LOC391322, MLLT3, MYC, NARF, p300-CBP, PLAC1, POLK, PREPL, RAB9A, RELA, SCAND1, SENP7, SLC5A7, STK16, STT3B, SULT1A2, TDP1, TMEM258, TMEM126A, TTR, UBC	19	Gene Expression, Cellular Development, Cell Cycle
6	ADAM10, ADAM17, Alp, ALPL, ALPPL2, Ap1, Collagen type IV, Erm, GLS, HSD11B1, Hsp70, IFN Beta, Ifn gamma, IL1, IL12 (complex), Interferon alpha, Laminin, MAP2K1/2, MAP4K2, Metalloprotease, MHC Class I (complex), Notch, P-TEFb, PADI4, PARP, PI3K (complex), Pro-inflammatory Cytokine, PSMD14, Sapk,	17	Cellular Development, Embryonic Development, Hematological System Development and Function

	sphingomyelinase, Tgf beta, thyroid hormone receptor, Tlr, TNKS, TNKS2		
7	15-LOX, 2'-fucosyllactose, 5-hydroxytryptophan, Anti-inflammatory Cytokine, BACE1, Calmodulin, chemokine, delta-aminolevulinic acid, FASTKD1, Focal adhesion kinase, FYN, ganglioside GD2, GSK3B, guanosine, IL12 (family), Il15r, IL36A, Immunoglobulin, myristic acid, Nfatc, NFkB (complex), NMDA Receptor, NR3C1, Orm, P38 MAPK, p85 (pik3r), PAFAH1B2, PARK2, platelet activating factor-C16, SENP6, SENP8, SRC (family), TARDBP, TRAF1-TRAF2-TRAF3, WISP3	13	Cell Morphology, Cellular Assembly and Organization, Nervous System Development and Function
8	15-LOX, Alpha tubulin, Ampa Receptor, c-Src, Calcineurin protein(s), calpain, CK1, CLK1, Collagen type I, Collagen(s), Dynein, EGFR, F3, Fibrinogen, Gap, HSD11B2, Integrin, JINK1/2, LCK, Lectin, Lfa-1, Lpa receptor, Mucin, NFAT (complex), P4HB, Pdgfr, PKC (α,β,γ,δ,ε,ι), PKC alpha/beta, Pp2b, PRKCB, PTPN1, RIN1, Sos, VAV, Wnt	13	Cell-To-Cell Signaling and Interaction, Tissue Development, Cancer
9	ADCY, Beta Arrestin, CHRM1, DRD1, DRD2, G protein, G protein alpha, G protein alphai, G protein beta gamma, Gi-coupled receptor, GNAI1, Gpcr, Gs-coupled receptor, Gαi/o, Il8r, Jnk, K Channel, Mmp, MTORC1, NPSR1, OPRD1, OPRK1, OPRM1, p70 S6k, PLC, Rac, Ras, Ras homolog, RGS12, Sfk, Shc, Sod, TAAR1, TSH, tubulin (complex)	12	Hereditary Disorder, Neurological Disease, Psychological Disorders
10	3alpha-hydroxysteroid dehydrogenase (A-specific), ACHE, AKR1B1, AKR1B10, AKR1C3, AKR1C4, AKR1C1/AKR1C2, Akt, ALDH1A1, Aldose Reductase, Atrial Natriuretic Peptide, BCR (complex), Cebp, HDL, HLA-DR, Igf, Igm, Ikb, IKK (complex), NADH or NADPH:quinone oxidoreductase, NFkB (family), Pdi, PI3K (family), PI3K p85, POLI, Ppp2c, PXR ligand-PXR-Retinoic acid-RXRα, retinal dehydrogenase, S6K1, SAA, trans-1,2-dihydrobenzene-1,2-diol dehydrogenase, tyrosine kinase, Ube3, Ubiquitin, Vla-4	11	Endocrine System Development and Function, Small Molecule Biochemistry, Energy Production
11	ALOX5, ALOX15, ALOX15B, AMPK, ATM/ATR, CDK6, Cyclin A, Cyclin D, Cyclin E, E2f, ERK, GADD45, GEM, glutathione peroxidase, Gm-csf, Histone H1, Hsp27, JAK, LDL, lipoxygenase, Mek, NADPH oxidase, NR1H4, Pdgf (complex), PDGF BB, PEPCK, Pka catalytic subunit, PLA2, PPARG, Rb, Rsk, Smad, STAT5a/b, TFIIH, THPO	11	Lipid Metabolism, Small Molecule Biochemistry, Molecular Transport

12	ACOT9, ANKRD13D, BCHE, CCDC42, COG1, CTDSP1, DPP7, EGFR, EMC3, EXOC6B, FBXO6, GALK1, GGH, GRAMD1A, HDHD3, KARS, KIAA0922, miR-625-5p (and other miRNAs w/seed GGGGGAA), MT1F, NAGLU, NUP85, OBP2B, RNASET2, RPF2, SAAL1, SIAE, STAT, STT3B, TBRG4, THADA, TM9SF2, TTC27, UBC, UROD, ZP3	6	Protein Degradation, Protein Synthesis, Hereditary Disorder
